# NMR-based serum metabolite and lipoprotein profiling for endometriosis across clinically relevant and physiological comparator settings: assessment of diagnostic utility and exploratory biological signals

**DOI:** 10.1186/s12916-026-04999-2

**Published:** 2026-06-17

**Authors:** Sisi Deng, André Koch, Bernhard Krämer, Claire Cannet, Yogesh Singh, Gyuntae Bae, James Sklut, Tony Reinsperger, Oscar Millet, Jürgen Andress, Lukas Schimunek, Christoph Trautwein

**Affiliations:** 1 Department of Preclinical Imaging and Radiopharmacy, Werner Siemens Imaging Center, University Hospital Tübingen, Tübingen, Germany; 2 Cluster of Excellence iFIT (EXC 2180) “Image Guided and Functionally Instructed Tumor Therapies”, University of Tübingen, Tübingen, Germany; 3 Core Facility Metabolomics, Faculty of Medicine, University of Tübingen, Tübingen, Germany; 4 M3 Research Center for Microbiome, Metabolome and Malignome, Faculty of Medicine, University of Tübingen, Tübingen, Germany; 5 Department of Women’s Health, University Hospital Tübingen, Tübingen, Germany; 6 Bruker BioSpin GmbH & Co. KG, Ettlingen, Germany; 7 Institute of Medical Genetics and Applied Genomics, University of Tübingen, Tübingen, Germany; 8 Precision Medicine and Metabolism Laboratory, CIC bioGUNE, BRTA, Derio, Bizkaia, Spain; 9 Consortium for Biomedical Research (CIBERCV, CIBERESP, CIBEREHD or CIBEROBN), Instituto de Salud Carlos III, Madrid, Spain

**Keywords:** Endometriosis, Metabolomics, Lipoproteins, ^1^H-NMR spectroscopy, Diagnostic modeling, Systems biology

## Abstract

**Background:**

Reliable non-invasive biomarkers for endometriosis remain unavailable in routine practice, and their translational value depends on performance in symptomatic referral populations rather than only against healthy controls. We evaluated Nuclear Magnetic Resonance (NMR)-based serum metabolite and lipoprotein profiling for endometriosis across clinically relevant and physiological comparator settings, alongside exploratory analyses of systemic biological variation.

**Methods:**

Blood serum samples from women with surgically confirmed endometriosis, symptomatic controls, and healthy volunteers underwent quantitative in vitro diagnostics research (IVDr) ^1^H-NMR-based metabolite and lipoprotein profiling. A subset also underwent cytokine profiling. Two diagnostic settings were prespecified: endometriosis versus symptomatic controls (primary) and endometriosis versus healthy volunteers (secondary). Baseline models included age and body mass index, while full models incorporated the IVDr metabolite-lipoprotein panel using elastic net regularization. Performance was assessed using fully nested repeated cross-validation and an independently processed temporal cohort. Exploratory analyses included covariate-adjusted group comparisons, weighted correlation network analysis, cytokine correlations, and paired pre-/post-operative comparisons.

**Results:**

In the primary symptomatic-control comparison, the IVDr panel did not improve diagnostic performance beyond age and body mass index (AUC 0.620 vs. 0.637 for baseline). Discrimination was substantially higher in the healthy-volunteer comparison (AUC 0.994 for the full model vs. 0.882 for baseline), but this pattern was not reproduced in the temporal cohort, where performance was poor in both comparator settings. Exploratory analyses showed that the clearest biological differences were concentrated in healthy-based contrasts, with lower amino acids, creatinine, lactic acid, and selected low-density lipoprotein (LDL) measures in endometriosis. Part of the amino-acid pattern was also present in symptomatic controls, whereas particularly LDL6 lipoprotein subfractions, appeared more restricted and were supported by lipoprotein-enriched network structure. Cytokine-cytokine correlations showed reproducible within-panel immune covariance, but no cross-domain correlations remained significant after false discovery rate correction.

**Conclusions:**

While NMR-based serum metabolite and lipoprotein profiling showed strong apparent discrimination against healthy volunteers, performance was limited in the clinically relevant symptomatic-control setting, underscoring the importance of comparator spectrum for translational biomarker evaluation. Exploratory analyses identified biologically informative serum patterns, particularly a more restricted lipoprotein-subclass LDL6 signal that warrants targeted replication in clinically representative and analytically harmonized studies.

**Supplementary Information:**

The online version contains supplementary material available at 10.1186/s12916-026-04999-2.

## Background

Endometriosis affects approximately 10% of women of reproductive age, corresponding to around 190 million globally [1, 2]. As one of the most common causes of infertility, endometriosis is associated with lower success rates in assisted reproductive technologies (ART) and adverse pregnancy outcomes, including preterm birth and placenta previa [3–5]. These reproductive consequences contribute to profound emotional distress, increased healthcare costs, and substantial socioeconomic burden [6].

Pain is the predominant symptom and can impair daily life, reduce productivity, and lead to frequent sick leave [7]. Chronic pain often leads to depression and impacts family well-being [8]. Despite a burden comparable to other chronic diseases, public and clinical awareness of endometriosis remains limited, and the condition continues to be underfunded and under-researched [9]. Symptoms are often mistaken for normal menstrual pain, contributing to diagnostic delays that worsen disease progression and treatment outcomes [9, 10].

Early diagnosis and effective treatment remain major clinical challenges [2, 11]. The European Society of Human Reproduction and Embryology (ESHRE) Guideline, widely recognized in Europe, recommends diagnosis based on clinical signs and symptoms in combination with pelvic examination, imaging, and histology when feasible [11]. However, negative imaging or histology does not fully exclude endometriosis, and reliable non-invasive biomarkers for routine diagnosis remain unavailable [12–14]. Consequently, ESHRE discourages biomarker use for routine diagnosis. Hormonal therapies may provide symptom relief, but efficacy is variable and side effects such as mood changes and irregular bleeding are common [15, 16]. In severe cases, surgical intervention becomes necessary [11, 16]. The ENZIAN classification describes deep infiltrating endometriosis (DIE) anatomically but does not correlate with symptom severity, limiting its use as a comprehensive disease staging system [17].

Surgical management often involves an initial diagnostic laparoscopy, followed by complete lesion resection (R0) when indicated [11]. Advanced-stage disease with adhesions can complicate surgery and increase the risk of complications such as intestinal fistulas [15, 18], often due to delayed diagnosis. High recurrence rates remain a challenge, with many patients requiring repeat surgeries [19]. In addition, women with endometriosis have been reported to face an increased risk of ovarian cancer, particularly in those with deep infiltrating endometriosis (DIE) [20], further underscoring the need for mechanistic studies.

Despite its clinical impact, the molecular mechanisms underlying endometriosis remain poorly understood. There is a pressing need for robust, non-invasive biomarkers to support early diagnosis, improve infertility management, and guide the development of new therapeutic strategies.

Metabolomics enables comprehensive profiling of small molecules and holds promise for identifying disease-related alterations. Proton nuclear magnetic resonance (^1^H-NMR) offers high reproducibility, quantitative accuracy, and suitability for large-scale, non-invasive clinical studies [21]. Its successful implementation in clinical monitoring programs in England and Australia further highlights its translational potential [22, 23].

We employed a multimodal approach integrating ^1^H-NMR metabolomics, cytokine profiling, and clinical data to characterize systemic alterations in endometriosis. Using serum samples measured via an in vitro diagnostics research (IVDr) NMR platform (Bruker BioSpin GmbH & Co. KG), we evaluated the performance of serum-based diagnostic models in both clinically relevant symptomatic-control and healthy-volunteer comparator settings, and examined their transportability in an independent temporal cohort. In parallel, we performed exploratory cross-sectional, network, cytokine-correlation, and longitudinal analyses to investigate coordinated metabolic and immune-related patterns. Our aim was not only to assess the translational potential of IVDr serum profiling, but also to examine, in parallel, how comparator spectrum and overlapping symptom biology shape diagnostic performance and exploratory biological interpretation in endometriosis research.

## Methods

### Study design

This study investigated serum metabolite, lipoprotein, and cytokine profiles in women evaluated for suspected endometriosis at the University Hospital Tübingen, Germany. The objectives were twofold: (1) to assess the diagnostic performance of serum-based prediction models, and (2) to explore disease-associated metabolic alterations.

Two diagnostic comparisons were defined: (1) Primary diagnostic comparison (D1), surgically confirmed endometriosis compared with symptomatic controls; (2) Secondary diagnostic comparison (D2), surgically confirmed endometriosis compared with healthy volunteers. The primary comparison reflects a clinically realistic diagnostic setting with overlapping symptoms, whereas the secondary comparison evaluates discrimination against healthy controls. Diagnostic model development used a fully nested repeated cross-validation framework to avoid overfitting and information leakage.

An independent temporal validation cohort was established from additional samples collected later and processed by a separate analytical team on the same IVDr NMR platform. This cohort was excluded from all model training and tuning.

In parallel, exploratory mechanistic analyses were performed using adjusted cross-sectional group comparisons and a paired pre- and post-operative endometriosis subset. These analyses were not used for diagnostic model development or validation.

**Fig. 1:**
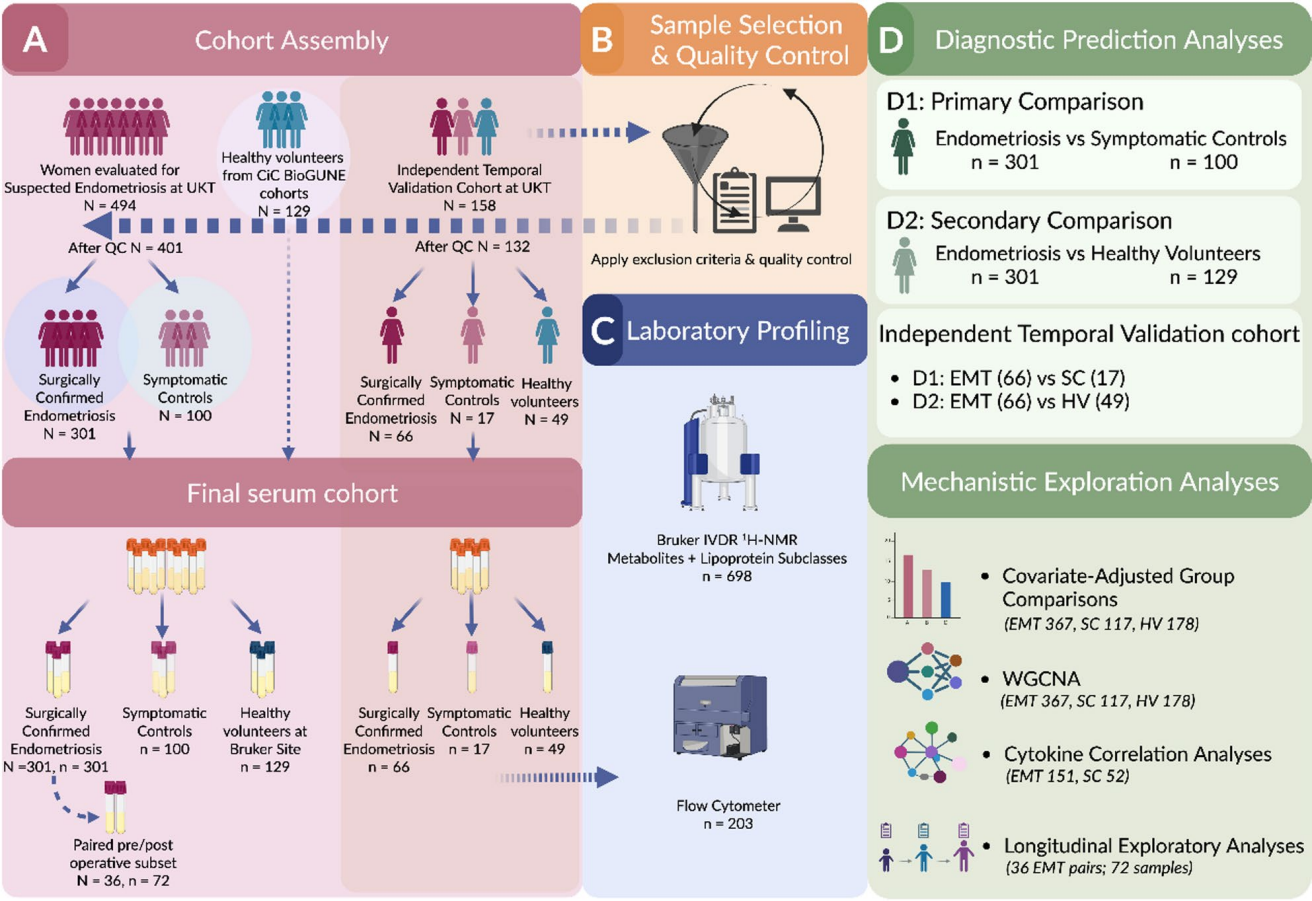
Overview of the study design and analytical workflow. **A** Cohort assembly after quality control, including surgically confirmed endometriosis cases, symptomatic controls, healthy volunteers, and an independent temporal validation cohort. A paired pre-/post-operative subset was retained for longitudinal exploratory analyses. **B** Sample selection and quality control. **C** Laboratory profiling by Bruker IVDr 1H-NMR for metabolites and lipoprotein subclasses, with a cytokine-measured subset analyzed by flow cytometry. **D** Downstream analyses, including diagnostic prediction for the primary and secondary comparisons, independent temporal validation, covariate-adjusted group comparisons, WGCNA, cytokine correlation analyses, and longitudinal exploratory analyses. Abbreviations: EMT, endometriosis; SC, symptomatic controls; HV, healthy volunteers; QC, quality control; WGCNA, weighted correlation network analysis; UKT, University Hospital Tübingen; CiC BioGUNE, Center for Cooperative Research in Biosciences

Patient enrollment and blood collection followed routine clinical workflow, and diagnosis was unknown at the time of sampling because surgical confirmation occurred later. Samples were therefore not intentionally ordered according to outcome. Serum samples were collected from women with clinically suspected endometriosis who visited the Endometriosis Center of University Hospital Tübingen (UKT) between March 2022 and March 2025. The overall study workflow, including cohort assembly, serum and cytokine profiling, and downstream diagnostic and exploratory analyses, is summarized in Fig. 1.

#### Endometriosis cohort

All endometriosis cases included in the final analyses were surgically confirmed. Diagnosis was based on clinical symptoms (e.g., dysmenorrhea, deep dyspareunia, dysuria, etc.), imaging (ultrasound and/or MRI), clinical exam, medical history, intraoperative findings, and histopathology where feasible [11]. Prior hormonal therapy was documented, acknowledging that it may obscure histological features [24]. In cases where minimal lesions were treated locally and insufficient tissue was available for histopathological confirmation, diagnosis was based on concordant clinical history, symptomatology, and intraoperative findings as assessed by an experienced gynecologic surgical team, in accordance with the ESHRE Guideline for Endometriosis [11]. A subset of endometriosis patients provided paired pre- and post-operative serum samples for longitudinal analysis, with samples collected within 1 week before surgery and 2–3 weeks postoperatively.

Exclusion criteria included: (1) Treatment for other gynecological conditions within one month before surgery; (2) History or presence of malignancy; (3) Missing essential clinical metadata; (4) Acute infection or antibiotic/probiotic use within one month pre-surgery.

Age, body mass index (BMI), and Enzian classification stages [17] were recorded and anonymized before analysis. Lifestyle and personal dietary information were incompletely documented and therefore not included in statistical adjustment.

#### Control cohorts

##### Symptomatic controls (D1, primary diagnostic comparison)

Symptomatic controls were women presenting with pelvic pain and/or gynecologic symptoms suggestive of endometriosis, but without surgical evidence of the disease.

##### Healthy volunteers (D2, secondary diagnostic comparison)

Healthy female volunteers were included as a secondary comparator group. A major subset of these samples was externally sourced through a research collaboration framework with Bruker BioSpin GmbH & Co. KG and derived from the AKRIBEA and OSARTEN cohorts at the Center for Cooperative Research in Biosciences (CiC BioGUNE), Spain, comprising individuals from the working general population of the Basque Country (OSARTEN, CEIC-E 16–114; AKRIBEA, CEIC-E 19 − 13) [25]. Samples were collected during scheduled medical check-ups according to standard operating procedures, aliquoted, and stored directly at − 80 °C. For NMR measurement, frozen serum samples were thawed for 30 min and prepared according to standardized Bruker IVDr NMR workflow (B.I. QUANT-PS™ and B.I. LISA™). Additional locally recruited healthy volunteers were included in the temporal comparison cohort, as described below.

Age and BMI were recorded for all control participants. Data were anonymized before analysis, and informed consent was obtained in compliance with regulations.

#### Independent temporal validation cohort

To evaluate the robustness of model performance beyond the development dataset, an independent validation cohort was assembled from additional serum samples collected during a later time period at the same hospital. Because a corresponding healthy-volunteer cohort was not available within the hospital-based biobank workflow, a locally recruited healthy-volunteer cohort from the University Hospital Tübingen was included as a secondary temporal spectrum comparison. This comparison was exploratory and was not intended to replace a clinically representative external validation benchmark. All validation samples were processed by a separate analytical team on the same IVDr platform.

This validation cohort was not used for model training, feature selection, or hyperparameter tuning. For temporal validation, preprocessing parameters and fitted models were derived from the development cohort only and then applied unchanged to the independent validation cohort. The validation analyses included: (1) a primary comparison (D1), endometriosis vs. symptomatic controls; and (2) a secondary validation comparison (D2), endometriosis vs. healthy volunteers.

### Ethical considerations

This study was approved by the Ethics Committee of the University of Tübingen, Germany (No. 265/2023BO2). Temporal healthy control samples were collected under a separate ethics approval from the same committee (No. 13/2007V). Written informed consent was obtained from all participants. Sample collection was non-interventional, and all data were anonymized in accordance with the Declaration of Helsinki, the General Data Protection Regulation (GDPR), and German data protection laws.

### Collection and storage of patient samples

Blood samples were processed within routine clinical workflow, generally at hospital admission under non-fasting conditions. Collection time-of-day was not standardized. Menstrual cycle phase and hormonal therapy exposure were variably documented and were not sufficiently complete for systematic statistical adjustment. Blood samples were centrifuged promptly after collection, and serum was aliquoted, transported on ice, and stored at − 80 °C in the Women’s Health Biobank until analysis. Samples underwent a single thaw at the time of NMR or cytokine measurement, and no additional freeze‑thaw cycles occurred. A metadata table was curated to capture available pre-analytical and technical variables, including sample identifiers, diagnostic group, site (UKT or externally sourced healthy cohort), and batch/run-order information where available. These variables were incorporated into technical sensitivity analyses. For externally sourced healthy volunteers, sample collection and storage procedures are described in Sect. 1.2.

### Cytokine quantification in serum

Serum cytokines were quantified using 25 µL samples and the LEGENDplex™ Human Inflammation Panel 1 (BioLegend, Cat. #740809), a bead-based multiplex assay targeting 13 cytokines. Samples were incubated with antibody-coated beads, followed by biotinylated detection antibodies and streptavidin-phycoerythrin. After washing, fluorescence signals were measured via flow cytometry and quantified using LEGENDplex™ software and standard curves.

### NMR-based serum metabolite and lipoprotein profiling

Serum metabolomics and lipoprotein profiling were performed using a Bruker Avance III HD 600 MHz NMR spectrometer with a 5 mm TXI probe, under standardized conditions per the Bruker IVDr protocol (AVANCE IVDr Methods v003). On the analysis day, serum was thawed and mixed 1:1 with pH 7.4 buffer (Bruker AH0622-10) containing D₂O, sodium monophosphate, TSP, and sodium azide [26]. After homogenization, 600 µL was transferred into 5 mm NMR tubes (Bruker Z168405), loaded into the SampleJet™ autosampler at 6 °C, and equilibrated in the probe head for 5 min. 1 H-NMR spectra were acquired using the NOESY pulse sequence and processed with Bruker TopSpin 3.6.1 and IVDr plug-ins. Metabolites and lipoprotein subfractions were quantified using B.I. QUANT-PS™ and B.I. LISA™, respectively. Spectral quality and instrument performance were monitored via B.I. BioBankQC™. Lipoprotein subclasses (e.g., VLDL-1 to VLDL-5) were classified by density, following established standards [23]. Detailed acquisition parameters are listed in Additional file 1: Table S1.

### Reproducibility and statistical analysis

All analyses were performed in R (version 4.4.3).

#### Reproducibility and sample size considerations

This study integrated diagnostic prediction modeling, exploratory mechanistic analyses, and longitudinal within-patient comparisons. As the primary objective was to evaluate predictive performance and reproducibility, model evaluation was based on fully nested resampling and independent temporal validation rather than on a single classical effect-size-based power calculation.

For exploratory mechanistic analyses, sample size considerations were estimated using the R *pwr* package. For case-control comparisons (Cohen’s d = 0.5, power = 0.95, α = 0.05), the estimated minimum sample size was 210 participants. For subgroup analyses (d = 0.25, power = 0.80), at least 159 participants across three groups were estimated to be required. These calculations were used to contextualize the exploratory group-comparison analyses rather than to define the prediction-modeling component of the study.

Fixed random seeds were specified within analysis scripts to improve reproducibility.

#### Data preprocessing

Preprocessing was performed separately for each analytical module according to its objective. For diagnostic prediction analyses, preprocessing was implemented within each training fold and then applied unchanged to the corresponding validation fold to prevent information leakage.

##### Diagnostic prediction analyses

Within each training partition, metabolite and lipoprotein features with ≥ 80% missing values were excluded. Remaining features were median-imputed. Age and BMI were also median-imputed when necessary. Metabolite and lipoprotein variables were then log-transformed using log1p and Pareto scaled (mean-centered and divided by the square root of the standard deviation), with scaling parameters derived from the training data only.

##### WGCNA

For network analysis, features with ≥ 80% missing values were excluded. Data were log-transformed and median imputed prior to network construction. To reduce redundancy, highly correlated variables (|r| > 0.95) were removed before module detection.

##### Cytokine analyses

Cytokines below the Lower Limit of Quantitation (LLOQ) were treated as missing according to assay-specified thresholds (Additional file 2: Table S2). Markers with ≥ 80% missingness were excluded. Remaining cytokines were imputed using MICE with predictive mean matching (m = 5, maxit = 5, seed = 202501). Imputation models included all retained cytokines as predictors. Downstream correlation analyses were performed using the first completed imputed dataset. Rubin’s rules were not applied, as the primary outputs were correlation structures rather than pooled regression coefficients.

##### Cross-sectional mechanistic and longitudinal exploratory analyses

For cross-sectional mechanistic comparisons and paired pre- and post-operative analyses, features with ≥ 80% missing values were excluded. Retained features were median-imputed and log1p transformed prior to regression-based group comparisons.

#### Confounder assessment

Age and BMI were prespecified covariates given their established association with circulating metabolic profiles and observed imbalance between comparison groups. Demographic balance was assessed using standardized mean differences (SMDs) and density plots for age and BMI across development and independent validation comparison panels. Potential technical influences, including site, batch, and run order, were evaluated separately in predefined technical sensitivity analyses (Sect.  6.6). Where appropriate, these factors were incorporated into adjusted models to assess their impact on diagnostic performance.

Because the secondary healthy-volunteer comparison showed marked imbalance in age and BMI, an additional sensitivity analysis was performed by pooling all available endometriosis cases and healthy volunteers across the development and temporal datasets. Samples with complete age and BMI data were matched 1:1 using nearest-neighbour propensity-score matching without replacement. Propensity scores were estimated by logistic regression with age and BMI as predictors, using a caliper of 0.2 on the standardized logit scale. Balance after matching was reassessed using standardized mean differences and density plots. The matched dataset was then re-evaluated as a secondary age/BMI-balanced endometriosis-versus-healthy analysis using the same nested cross-validation framework.

#### Predictive modeling framework

Diagnostic performance was evaluated using a fully nested repeated cross-validation framework to minimize optimism bias and prevent information leakage. The outer loop consisted of 5-fold cross-validation repeated five times (5-fold × 5 repeats) to obtain robust performance estimates, while the inner loop was used exclusively for hyperparameter tuning. Because the primary objective was unbiased evaluation of model performance under nested resampling, the analyses focused on repeated training and validation across data splits rather than on deriving a single fixed model for immediate clinical deployment.

Two diagnostic comparison settings were prespecified and within each setting, a baseline and a full model were evaluated. For the primary model, the outcome was surgically confirmed endometriosis versus symptomatic controls without surgical evidence of endometriosis; for the secondary model, the outcome was surgically confirmed endometriosis versus healthy volunteers. The baseline model consisted of a logistic regression including age and BMI. The full model incorporated age, BMI, and the complete IVDr NMR metabolite and lipoprotein feature panel using elastic net regularization, which allows simultaneous coefficient shrinkage and feature selection in high-dimensional settings. Model tuning was conducted within the internal training partitions only. Regularization parameters were selected within each training fold using internal cross-validation. The final model for each outer split was then evaluated on its corresponding validation fold. Feature selection patterns across resampling iterations were examined descriptively.

#### Performance metrics and clinical utility

Model performance was evaluated with respect to discrimination, calibration, and potential clinical utility. Discrimination was quantified using the area under the receiver operating characteristic curve (AUC), Brier score, and sensitivity and specificity at the Youden-optimal threshold.

For the nested cross-validation analyses, performance metrics were summarized across outer validation folds to provide an unbiased estimate of model performance. Uncertainty intervals were derived from the empirical distribution of outer-fold performance estimates. These are reported as 95% empirical intervals. The incremental contribution of the IVDr NMR metabolite-lipoprotein panel beyond the baseline demographic model (age and BMI) was quantified as the change in AUC and Brier score.

Calibration was assessed using reliability curves comparing predicted and observed event probabilities, with 95% confidence intervals for observed proportions.

To evaluate potential clinical usefulness, decision-curve analysis was performed across a range of clinically plausible threshold probabilities (0.05–0.50), estimating the net benefit of applying each model in practice. For the primary symptomatic-control comparison, positive and negative predictive values were additionally translated to assumed disease prevalences representative of referral settings (20–50%). Exploratory incremental value measures and stratified performance across age and BMI categories were also examined.

#### Independent validation and technical sensitivity analyses

An independent temporal validation cohort was assembled from serum samples collected during a later time period and processed by a separate analytical team using the same IVDr NMR platform within UKT. These samples were not used for model development, preprocessing parameter estimation, or hyperparameter tuning. Models derived from the development cohort were applied unchanged to the independent validation cohort to assess reproducibility of performance estimates.

Technical metadata, including acquisition site, analytical batch, and run order where available, were curated in a structured dataset. The primary endometriosis vs. symptomatic-controls comparison consisted of samples processed within a shared institutional workflow, allowing meaningful evaluation of technical covariates.

In contrast, the development-stage secondary comparison between endometriosis and healthy volunteers included externally processed healthy samples that did not share a common run sequence with local samples. Because site and batch were structurally confounded with diagnostic group, technical adjustment was not considered interpretable in this setting. Accordingly, the results were interpreted as a secondary spectrum comparison rather than as a primary clinical validation.

For the primary comparison, predefined technical sensitivity analyses were conducted to evaluate potential batch and run-order effects within the shared institutional workflow. Variance structure and possible clustering by batch or run order were examined using principal component analysis. Diagnostic models were then re-estimated with batch and run-order included as covariates within the same nested cross-validation framework to assess their impact on performance. In addition, an empirical Bayes batch correction (*ComBat*) was applied within cross-validation splits as an unsupervised sensitivity analysis prior to model fitting. Because batch correction modifies feature distributions within cross-validation splits, this analysis was interpreted as a robustness assessment rather than as the primary unbiased performance estimate. The Bruker IVDr platform incorporates standardized instrument and spectral quality-control procedures. However, no study-specific longitudinal quality control (QC) indicator samples were available to formally track analytical drift across the full measurement period. This limitation was considered in the technical assessment.

#### Mechanistic exploration

Exploratory mechanistic analyses were conducted independently of the diagnostic modeling framework and were not used for diagnostic model development, tuning, or validation.

A combined cross-sectional dataset including endometriosis, symptomatic controls, and healthy volunteers was analyzed using feature-wise covariate-adjusted linear regression models. For these analyses, curated technical metadata (including site and, where applicable, batch and run order) were incorporated to account for potential pre-analytical and analytical influences. Healthy volunteers included both locally collected and externally sourced CiC BioGUNE healthy samples in this exploratory module [25].

After preprocessing (Sect.  6.2), prespecified regression models were fitted for omnibus three group comparisons and for targeted pairwise contrasts. Age and BMI were included as biological covariates in all models. Site was included for cross-site comparisons, while batch and run order covariates were incorporated in local-only contrasts where they were technically meaningful. Run order was not included in cross-site contrasts because instrument sequences were not shared across acquisition settings. Technical covariates lacking variability within a given subset were omitted to ensure model stability.

For each feature, adjusted effect estimates (on the transformed scale), p values, and Benjamini-Hochberg false discovery rate (FDR) adjusted q values were computed. FDR correction was applied separately within metabolite and lipoprotein feature families for each contrast.

#### Network and cytokine-metabolite correlation analyses

Exploratory network and immune-metabolic analyses were conducted independently of the diagnostic modeling framework.

##### Weighted correlation network analysis (WGCNA)

The primary WGCNA analysis used the full available dataset, including healthy volunteers, symptomatic controls, and surgically confirmed endometriosis subgroups classified as non-deep infiltrating endometriosis (NDIE) and deep infiltrating endometriosis (DIE). To assess whether module structure remained robust in more biologically homogeneous subgroup comparisons, module preservation analyses were then performed after excluding symptomatic controls, with preservation evaluated across the Healthy volunteers, NDIE, and DIE subsets.

Features with ≥ 80% missingness were excluded. Data were log-transformed, median-imputed, and highly correlated variables (|r| > 0.95) were pruned to reduce redundancy prior to network construction. A signed network was constructed, and module eigengenes were correlated with clinical traits. Multiple testing across module trait associations was controlled using Benjamini-Hochberg FDR.

Sensitivity analyses included module preservation across Healthy volunteers, NDIE, and DIE subsets after exclusion of symptomatic controls, as well as re-analysis using an unsigned network framework.

##### Cytokine correlation analyses

Cytokine associations were evaluated in two prespecified cohorts: (1) an endometriosis-only cohort as the primary exploratory analysis, and (2) a broader cohort including endometriosis and symptomatic controls as a sensitivity comparison. After preprocessing (Sect.  6.2), cytokine, metabolic and lipoprotein features were aligned by sample ID. Spearman rank correlations were computed to evaluate cytokine-metabolite, cytokine-lipoprotein, and cytokine-cytokine relationships.

FDR was controlled using the Benjamini-Hochberg procedure separately within each predefined test family. Only associations surviving within-family FDR correction were considered statistically significant in the primary analysis. Associations meeting nominal significance (raw *p* < 0.05) but not FDR criteria were reported in the additional files and explicitly labeled as exploratory.

#### Longitudinal exploratory comparison

A paired pre- and post-operative subset of endometriosis patients was analyzed as an exploratory longitudinal module to complement cross-sectional mechanistic findings. This analysis was not designed to evaluate diagnostic performance. Only patients with one pre-operative and one post-operative serum sample were included. After preprocessing (missingness filtering, median imputation, and log transformation), feature-wise within-patient regression models were fitted to estimate post-operative changes relative to baseline.

Models were specified using patient-level fixed effects to assess changes within the same individual, thereby controlling for stable inter-individual characteristics. As comparisons were conducted within patients over a short time interval, age and BMI were not included as additional covariates. A predefined technical sensitivity analysis incorporated batch and run-order covariates when these were estimable within the subset. Where technical variables lacked variation, they were omitted to preserve model identifiability. Benjamini–Hochberg FDR correction was applied separately within metabolite and lipoprotein feature families.

## Results

### Cohort definition

#### Patient clinical characteristics

A total of 530 serum samples from 494 participants with clinically suspected endometriosis were initially collected in the hospital-based cohort (Fig. 1). After quality control and exclusion of samples not meeting predefined criteria, preoperative serum samples were retained for cross-sectional analyses, comprising 301 surgically confirmed endometriosis cases and 100 symptomatic controls. All endometriosis cases included in the analyses were surgically confirmed by intraoperative findings with histopathological confirmation where feasible. A subset of 36 patients provided paired pre- and post-operative samples for longitudinal exploratory analysis. Additionally, 129 externally sourced healthy female volunteers from the CiC BioGUNE AKRIBEA/OSARTEN cohorts [25], processed under standardized Bruker IVDr NMR protocols, were included as a secondary comparator group.

Symptomatic controls represented patients presenting with overlapping pelvic pain or gynecologic symptoms but without surgical evidence of endometriosis.

Two diagnostic comparison cohorts were defined. The primary comparison consisted of endometriosis (*n* = 301) versus symptomatic controls (*n* = 100), reflecting a clinically realistic differential diagnostic setting. The secondary comparison consisted of endometriosis (*n* = 301) versus healthy volunteers (*n* = 129), representing a physiological contrast.

An independent temporal validation cohort was assembled from separately processed samples and was not used for model development or tuning. This cohort initially included 109 women with clinically suspected endometriosis and 49 healthy volunteers (Fig. 1). After quality control and exclusion of samples not meeting predefined criteria, 66 surgically confirmed endometriosis cases and 17 symptomatic controls were retained. The corresponding validation comparisons were endometriosis (*n* = 66) versus symptomatic controls (*n* = 17) for the primary validation analysis, and endometriosis (*n* = 66) versus healthy volunteers (*n* = 49) for the secondary validation analysis.

#### Covariate balance

SMDs were calculated to assess imbalance in age and BMI between groups. In the primary symptomatic comparison, modest imbalance was observed for age (absolute SMD = 0.35) and BMI (absolute SMD = 0.27). In contrast, the secondary healthy-volunteer comparison showed substantial age imbalance (absolute SMD = 1.84) and moderate BMI imbalance (absolute SMD = 0.39). These differences are compatible with the strong baseline discriminatory performance of age and BMI in the healthy-volunteer comparison. Density plots illustrating age and BMI distributions across development and independent validation panels are provided in Additional file 3: Figure S1. In an additional propensity score-matched age/BMI-balanced subgroup for the endometriosis-versus-healthy comparison, post-matching density plots showed improved covariate balance (Additional file 3: Figure S1E).

### Diagnostic prediction analyses

#### Primary diagnostic analysis: endometriosis vs. symptomatic controls

##### Baseline model

**Fig. 2:**
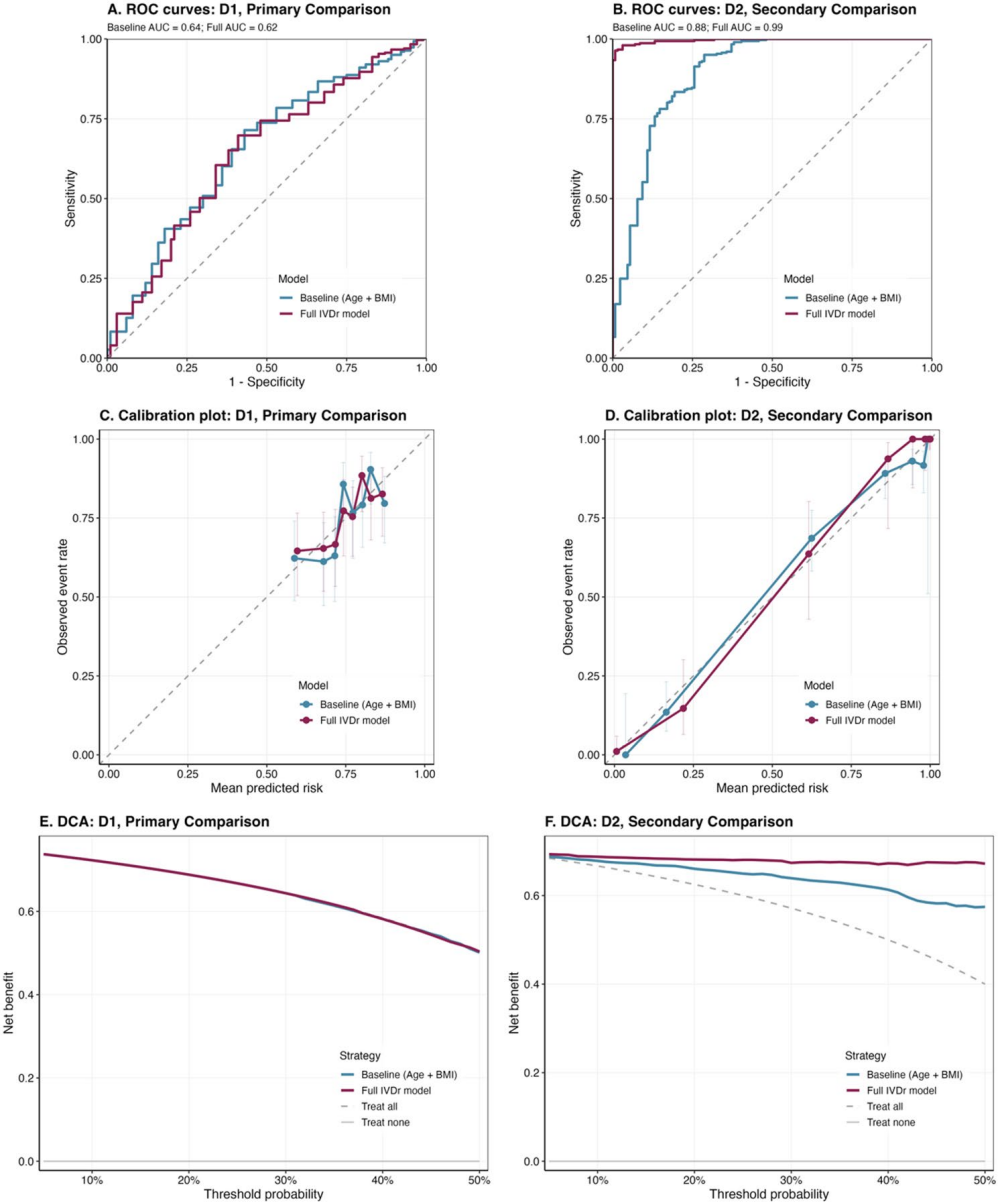
Internal diagnostic performance in the primary symptomatic-control comparison and the secondary healthy-volunteer comparison. **A**,** B** ROC curves for D1 and D2, respectively. **C**,** D** Calibration plots for D1 and D2, respectively. **E**,** F** Decision-curve analyses for D1 and D2, respectively. Overall, the full IVDr model showed no clear improvement over the baseline model in the primary symptomatic-control comparison, whereas stronger apparent discrimination and clinical utility were observed in the secondary healthy-volunteer comparison. Abbreviations: AUC, area under the curve; BMI, body mass index; IVDr, in vitro diagnostics research

In the primary diagnostic comparison between endometriosis and symptomatic controls, the baseline logistic regression model including age and BMI showed only modest discrimination within the fully nested repeated cross-validation framework (5-fold × 5 repeats) (Fig. 2A). The model achieved an AUC of 0.637 (95% empirical interval 0.530–0.753) with a Brier score of 0.181. At the Youden-optimal threshold, sensitivity was 0.688 and specificity was 0.520. Overall, routinely available demographic variables provided limited separation between surgically confirmed endometriosis and symptomatic patients without surgical evidence of disease.

##### Full IVDr panel model

Adding the full IVDr metabolite-lipoprotein panel to age and BMI using elastic net regularization did not improve performance in this symptomatic-control setting (Fig. 2A). The full model achieved an AUC of 0.620 (95% empirical interval 0.526–0.757) and a Brier score of 0.182, with sensitivity and specificity of 0.658 and 0.532 at the Youden optimal threshold. The incremental discrimination relative to the baseline model was small and negative (ΔAUC = -0.018; 95% empirical interval − 0.069 to 0.033), indicating no evidence of clinically meaningful gain from adding high-dimensional IVDr features beyond age and BMI in this clinically relevant comparator spectrum.

##### Calibration and clinical utility

Calibration analysis showed similar agreement between predicted and observed probabilities for both baseline and full models (Fig. 2C), with no clear improvement after addition of the IVDr panel.

To contextualize performance in clinically relevant referral settings, positive predictive values (PPVs) and negative predictive values (NPVs) were translated across assumed disease prevalences of 20%, 30%, 40%, and 50%. Predictive values were similar between models at all prevalence levels. At an assumed prevalence of 20%, PPV/NPV were 0.264/0.870 for the baseline model and 0.260/0.861 for the full model. At 50% prevalence, PPV/NPV were 0.589/0.625 and 0.584/0.608, respectively. These findings indicate that incorporation of the IVDr metabolite-lipoprotein panel did not meaningfully alter post-test probabilities in a symptomatic population.

Decision curve analysis across threshold probabilities from 0.05 to 0.50 showed largely overlapping net-benefit curves (Fig. 2E). At clinically relevant thresholds of 0.10, 0.20, 0.30, and 0.40, the difference in net benefit between models was zero. Incremental discrimination metrics were likewise minimal or unfavorable. The sample-level summary showed a ΔAUC of -0.014, a continuous net reclassification improvement (NRI) of 0.023, and an integrated discrimination improvement (IDI) of -0.005. Collectively, these findings consistently indicate that, in a symptomatic referral population, adding the IVDr panel did not provide clinically meaningful improvement beyond age and BMI alone.

##### Stratified performance by age and BMI quintiles

To assess whether performance varied across demographic strata, discrimination was examined within age and BMI quintiles. Performance remained modest across all subgroups for both models, and no stratum demonstrated material improvement with inclusion of the IVDr panel. Across age quintiles, baseline AUCs ranged from 0.489 to 0.660, while full-model AUCs ranged from 0.478 to 0.603. Across BMI quintiles, baseline AUCs ranged from 0.434 to 0.661 and full-model AUCs ranged from 0.418 to 0.638. A total of 52 samples were excluded from the BMI quintile analysis because of missing BMI. These findings suggest that the limited discrimination observed in the primary symptomatic comparison was not driven by a specific demographic subgroup.

#### Secondary diagnostic analysis: endometriosis vs. healthy volunteers

##### Baseline model

In contrast to the symptomatic-control setting, discrimination was substantially higher when comparing surgically confirmed endometriosis cases with healthy female volunteers (Fig. 2B). Within the fully nested repeated cross-validation framework, the baseline logistic regression model including age and BMI achieved an AUC of 0.882 (95% empirical interval 0.816–0.948) and a Brier score of 0.104. At the Youden-optimal threshold, sensitivity was 0.894 and specificity was 0.724. These results indicate clearer separation between disease and healthy physiological states than observed in the clinically overlapping symptomatic cohort.

##### Full IVDr NMR panel model

Incorporation of the full IVDr metabolite-lipoprotein panel using elastic net regularization further increased discrimination in this healthy-volunteer comparison (Fig. 2B). The full model achieved an AUC of 0.994 (95% empirical interval 0.976–1.000) with a Brier score of 0.027, and sensitivity and specificity of 0.975 and 0.932, respectively. Relative to the baseline model, the incremental change in discrimination was + 0.112 (95% empirical interval 0.047–0.177). Calibration analysis showed close agreement between predicted and observed probabilities, and decision curve analysis showed higher net benefit across the evaluated threshold range compared with the baseline model (Fig. 2D, F).

#### Impact of control spectrum

These findings should be interpreted in the context of comparator spectrum. While the IVDr NMR panel demonstrated very high discrimination when evaluated against healthy volunteers, no incremental benefit was observed in the symptomatic-control analysis. This contrast highlights the strong influence of control selection on apparent model performance. Discrimination against healthy individuals may therefore overestimate diagnostic utility in real-world referral settings, where patients typically present with overlapping symptoms rather than health.

### Independent temporal validation and technical sensitivity

#### Independent temporal validation

To assess model robustness beyond the development dataset, performance was evaluated in an independent cohort collected at a later time period and processed by a separate analytical team using the same IVDr NMR platform. This cohort was not used in model training or tuning.

In the independent endometriosis (*n* = 66) versus symptomatic-control comparison (*n* = 17), discriminatory performance was limited. The baseline model including age and BMI yielded an AUC of 0.510 (95% CI 0.368–0.651), and the full IVDr NMR panel model achieved an AUC of 0.500 (95% CI 0.357–0.643), consistent with near-chance classification.

In the independent endometriosis (*n* = 66) versus healthy volunteers (*n* = 49) comparison, the baseline age + BMI model demonstrated poor discrimination (AUC = 0.325, 95% CI 0.225–0.425). The full metabolite-lipoprotein panel did not reproduce the high discrimination observed in internal analysis, with an AUC of 0.468 (95% CI 0.360–0.577). Thus, the high discrimination observed in the internal healthy-volunteer comparison was not reproduced in the independent temporal cohort.

**Fig. 3:**
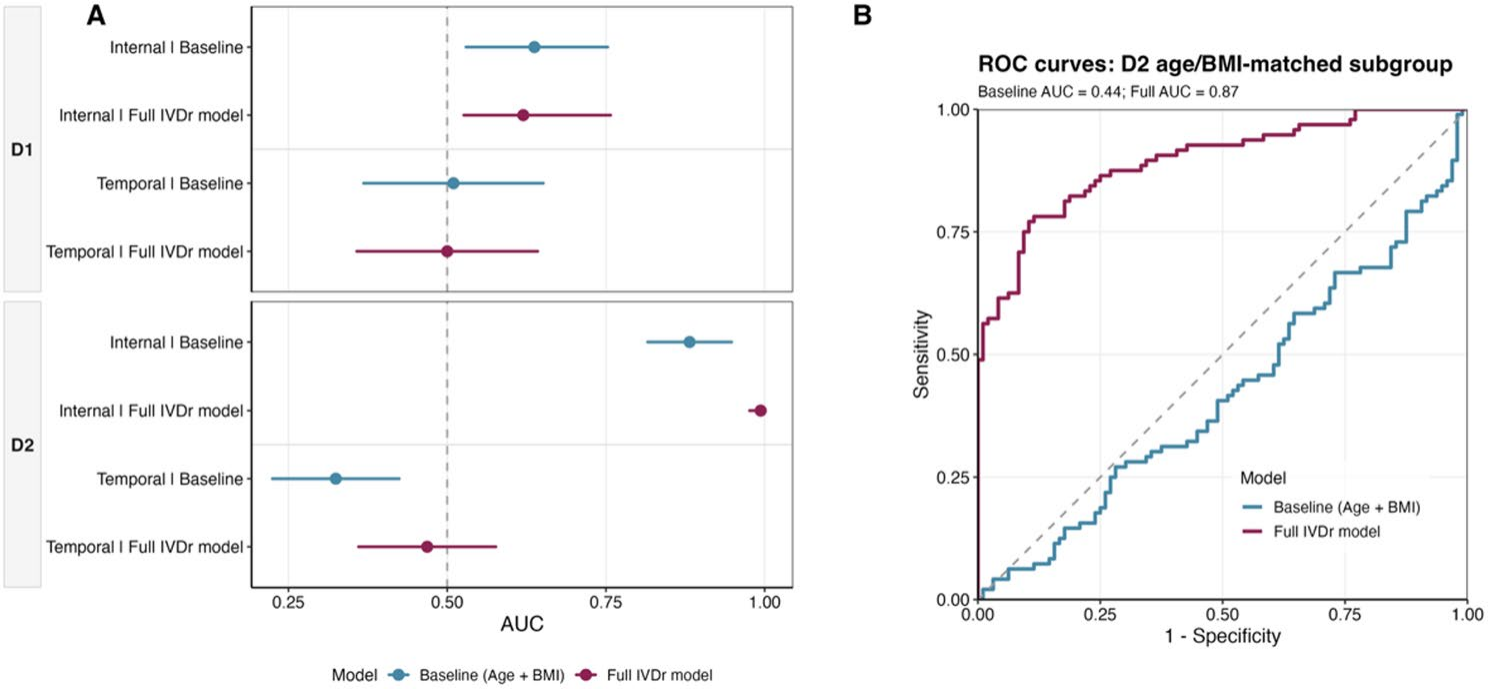
Validation summary and additional age/BMI-matched sensitivity analysis for the endometriosis-versus-healthy comparison. **A** Points indicate AUC estimates for the baseline model (age + BMI) and the full IVDr model in the primary symptomatic-control comparison (D1) and the secondary healthy-volunteer comparison (D2), shown for both internal and independent temporal analyses. Error bars represent 95% empirical intervals for internal nested cross-validation estimates and 95% confidence intervals for the temporal cohort. Overall, the panel shows limited incremental value of the IVDr panel in D1, strong apparent internal discrimination in D2, and marked attenuation of performance in temporal validation, particularly in the healthy-volunteer comparison. **B** Receiver operating characteristic (ROC) curves from an additional age/BMI-matched sensitivity subgroup for D2, derived by propensity-score matching after pooling all available endometriosis cases and healthy volunteers across the development and temporal datasets. In this matched subgroup, the baseline age + BMI model showed near-chance discrimination, whereas the full IVDr model retained substantially higher discrimination. This pattern suggests that part of the original D2 separation was influenced by age/BMI imbalance, while also supporting the presence of a residual metabolite-lipoprotein signal after balancing these measured covariates. Abbreviations: AUC, area under the curve; BMI, body mass index; IVDr, in vitro diagnostics research

Overall, external validation performance was poor across both comparison settings, indicating limited reproducibility of the internally derived models in this independently processed cohort. A summary of internal and temporal validation performance across the primary and secondary comparator settings is shown in Fig. 3A.

To further probe the marked discrepancy between the strong internal performance and poor temporal performance in the endometriosis-versus-healthy comparison, we performed an additional age/BMI-matched sensitivity analysis by pooling all available endometriosis cases and healthy volunteers across the development and temporal datasets. After 1:1 propensity-score matching, 96 endometriosis cases and 96 healthy volunteers were retained, and age/BMI imbalance was markedly reduced (absolute SMD 0.009 for age and 0.067 for BMI). In this matched dataset, the baseline age + BMI model showed near-chance discrimination (AUC 0.442, 95% empirical interval 0.338–0.544), whereas the full IVDr model retained substantially higher discrimination (AUC 0.874, 95% empirical interval 0.769–0.962; ΔAUC 0.432, 95% empirical interval 0.261–0.571; Fig. 3B). These findings suggest that part of the original endometriosis-versus-healthy separation was attributable to age/BMI imbalance, while also supporting the presence of an additional metabolite-lipoprotein signal after balancing these measured covariates.

#### Technical structure and sensitivity analyses

Principal component analysis was performed to examine potential technical structure associated with batch and run order (Additional file 3: Figure S2). For most principal components, variance explained by these technical factors was low (R² range, 0.004–0.021). One component (PC9) demonstrated a stronger technical association (R² = 0.115; p for batch = 9.44 × 10⁻¹⁰; p for run order = 4.59 × 10⁻⁴), indicating detectable but localized technical contribution within the feature space.

To evaluate whether technical factors materially influenced diagnostic performance in the primary symptomatic comparison, models were refitted with batch and run order covariates included. Discriminatory performance remained modest (batch-adjusted baseline AUC = 0.620; batch-adjusted full-model AUC = 0.605). A ComBat sensitivity analysis yielded similar results (ComBat-adjusted full-model AUC = 0.615).

Overall, although measurable technical structure was detectable in a subset of features, adjustment for batch and run order did not materially alter the diagnostic conclusions in the symptomatic comparison.

### Mechanistic exploration

These analyses were exploratory and independent of the diagnostic model development framework. Feature-wise covariate-adjusted linear models were fitted across healthy volunteers (*n* = 178), symptomatic controls (*n* = 117), and endometriosis cases (*n* = 367), with adjustment for age, BMI, and site. FDR was controlled separately within metabolite and lipoprotein feature families.

#### Covariate-adjusted three-group comparison

**Fig. 4:**
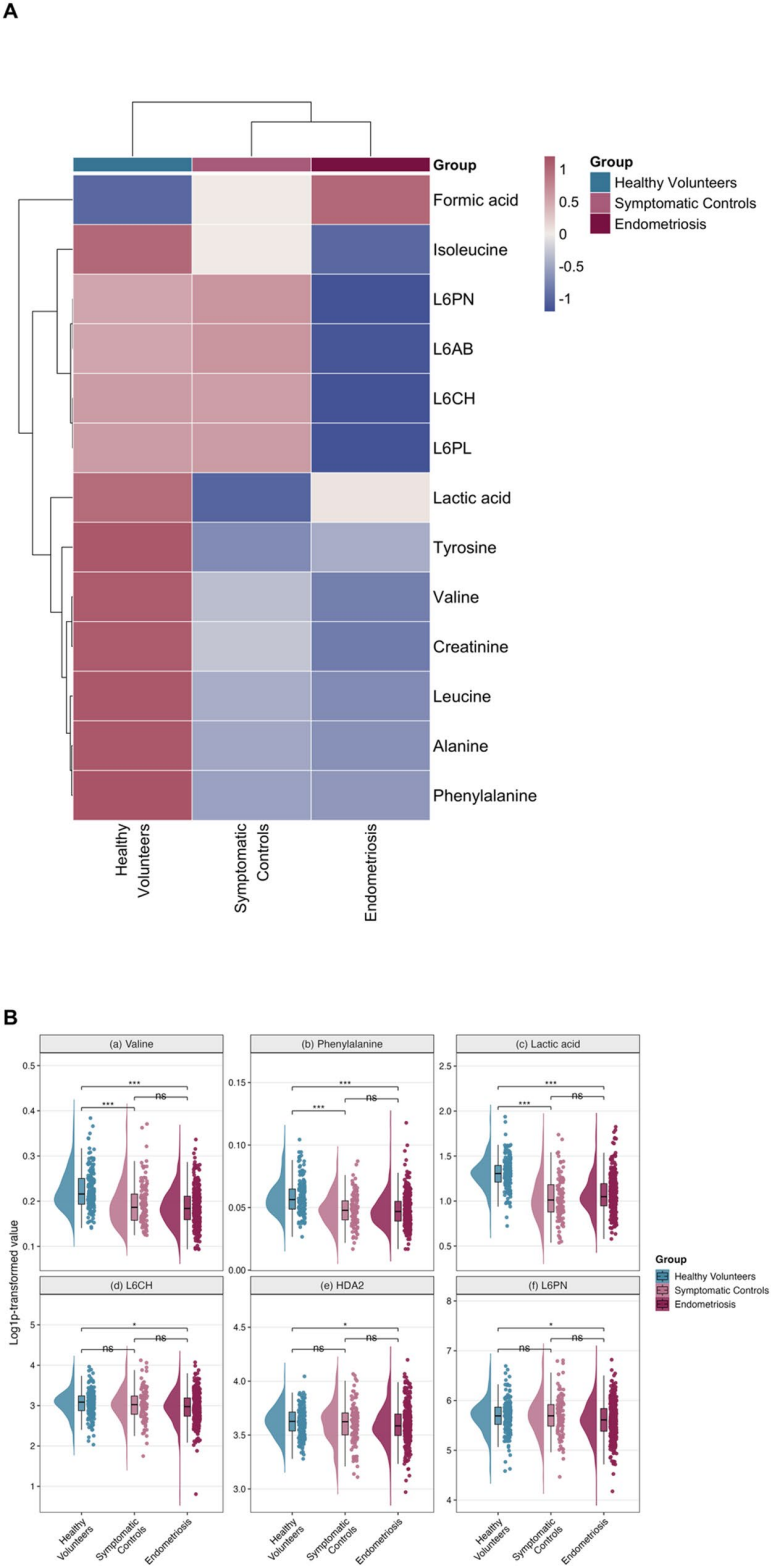
Covariate-adjusted group differences across healthy volunteers, symptomatic controls, and endometriosis. **A** Heatmap of the 13 metabolite and lipoprotein features that remained significant after Benjamini–Hochberg FDR correction in the covariate-adjusted three-group comparison. Displayed values represent standardized group-level patterns. **B** Representative raincloud plots for selected features (valine, phenylalanine, lactic acid, L6CH, HDA2, and L6PN) across healthy volunteers, symptomatic controls, and endometriosis. The plots illustrate shared amino-acid and lactic-acid differences across healthy-based contrasts, whereas lipoprotein-related differences were more apparent in the endometriosis-versus-healthy comparison. Statistical annotations indicate pairwise group differences after covariate adjustment. Asterisks denote FDR-adjusted significance levels (*FDR < 0.05, **FDR < 0.01, ***FDR < 0.001), and ns indicates a non-significant comparison. Abbreviations: FDR, false discovery rate; ns, not significant

In the three-group comparison, 13 features remained significant after FDR correction, including 9 metabolites and 4 lipoprotein measures. The significant metabolites were alanine, creatinine, formic acid, isoleucine, lactic acid, leucine, phenylalanine, tyrosine, and valine. The significant lipoprotein features were L6AB, L6CH, L6PL, and L6PN. The covariate-adjusted three-group heatmap and representative feature distributions are shown in Fig. 4.

#### Endometriosis vs. Healthy Volunteers

In the covariate-adjusted comparison between endometriosis and healthy volunteers, 16 features remained significant after FDR correction, comprising 9 metabolites and 7 lipoprotein measures. The significant metabolites were alanine, creatinine, formic acid, isoleucine, lactic acid, leucine, phenylalanine, tyrosine, and valine. The significant lipoprotein features were H3CH, HDA2, L6AB, L6CH, L6PL, L6PN, and TPA2. Effect estimates for these healthy-based differences are summarized in Additional file 3: Figure S3. Representative feature distributions are shown in Fig. 4B, and complete three-group raincloud plots for all 16 unique features significant in at least one adjusted mechanistic contrast are provided in Additional file 3: Figure S4.

#### Endometriosis vs. symptomatic controls

No metabolites or lipoprotein features remained significant after FDR correction in the comparison between endometriosis and symptomatic controls.

#### Symptomatic controls vs. healthy volunteers

Alanine, isoleucine, lactic acid, leucine, phenylalanine, tyrosine, and valine remained FDR significant in the comparison between symptomatic controls and healthy volunteers. No lipoprotein features reached FDR significance in this contrast. Corresponding effect estimates are shown in Additional file 3: Figure S3.

### Exploratory network and cytokine-metabolite correlation analyses

#### Weighted correlation network analysis

**Fig. 5:**
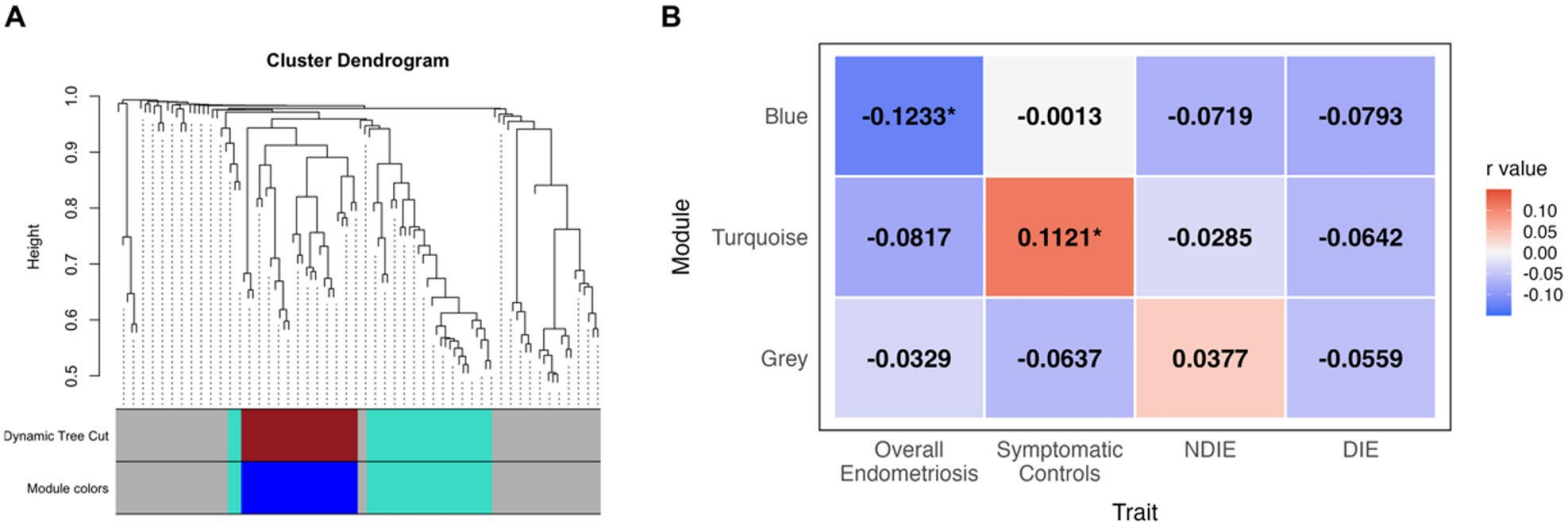
Weighted correlation network analysis of metabolite and lipoprotein features. **A** Hierarchical clustering dendrogram and module assignment from the weighted Gene Co-expression Network Analysis (WGCNA) network, showing two non-grey modules (blue and turquoise) and the remaining features assigned to the grey module. **B** Module–trait correlation heatmap for the signed network. Values indicate Pearson correlation coefficients between module eigengenes and predefined clinical traits. After Benjamini-Hochberg FDR correction, the blue module was negatively associated with overall endometriosis status (NDIE + DIE vs. healthy participants + symptomatic controls), whereas the turquoise module was positively associated with symptomatic-control status (symptomatic controls vs. all remaining samples). Effect sizes were modest, indicating subtle but detectable module-level variation across clinical groupings. Asterisks indicate FDR < 0.05. Abbreviations: DIE, deep infiltrating endometriosis; NDIE, non-deep infiltrating endometriosis

Weighted correlation network analysis was performed using the WGCNA framework on metabolite and lipoprotein features in the full cohort (*n* = 662), including healthy volunteers (*n* = 178), symptomatic controls (*n* = 117), and surgically confirmed endometriosis (*n* = 367), further classified as non-deep infiltrating endometriosis (NDIE, *n* = 68) and deep infiltrating endometriosis (DIE, *n* = 299). After excluding features with high missingness and pruning highly correlated variables to reduce redundancy, a signed network was constructed from 108 retained features. Two non-grey modules were identified (blue, 26 features; turquoise, 31 features), with the remaining 51 features assigned to the grey module (Fig. 5A, Additional file 2: Table S3).

Module-trait associations were evaluated using predefined clinical traits. After Benjamini-Hochberg FDR correction, two module-trait correlations remained statistically significant (Fig. 5B). The blue module eigengene was negatively associated with overall endometriosis status, defined as pooled endometriosis cases (NDIE and DIE) versus pooled non-endometriosis samples (healthy volunteers and symptomatic controls) (*r* = − 0.123, q = 0.0178). By contrast, the turquoise module eigengene was positively associated with the symptomatic-control contrast, defined as symptomatic controls versus all remaining samples (healthy volunteers, NDIE and DIE) (*r* = 0.112, q = 0.0232). Effect sizes were modest, indicating subtle module-level variation across clinical groupings.

The blue module was composed predominantly of lipoprotein subclass variables, whereas the turquoise module included both lipoprotein measures and several amino acids, including isoleucine, leucine, threonine, and valine. Full module membership is provided in Additional file 2: Table S3.

Sensitivity analyses suggested broadly similar module structure. After excluding symptomatic controls, module preservation analyses across healthy volunteers, NDIE, and DIE subsets showed preservation statistics above conventional descriptive thresholds (all Zsummary > 5). An unsigned-network sensitivity analysis yielded a broadly similar module structure, although some features were reassigned at module boundaries.

#### Cytokine correlation analyses

Exploratory cytokine correlation analyses were conducted in participants with cytokine measurements, including an endometriosis-only cohort (*n* = 151) and a sensitivity cohort comprising endometriosis plus symptomatic controls (*n* = 203). After Benjamini-Hochberg FDR correction, no cytokine-metabolite or cytokine-lipoprotein correlations remained statistically significant in either cohort (0/468 and 0/1,456 tests, respectively). Nominal associations (raw *p* < 0.05) are reported in Additional file 4: Table S4A as exploratory findings, including 24 cytokine-metabolite and 66 cytokine-lipoprotein pairs in the endometriosis-only cohort, and 25 cytokine-metabolite and 44 cytokine-lipoprotein pairs in the sensitivity cohort.

By contrast, cytokine-cytokine correlations showed a consistent within-panel immune correlation structure, with 48/78 FDR-significant cytokine pairs in the endometriosis-only cohort and 58/78 in the sensitivity cohort (Additional file 5: Table S4B). Overall, the findings support detectable cytokine co-variation within the measured panel but do not provide FDR-robust evidence for coupling between cytokines and serum metabolite or lipoprotein features in this exploratory subgroup.

### Longitudinal exploratory analyses

A paired subset comprising 36 patients with complete pre- and post-operative samples (72 total samples) was analyzed to evaluate short-term within-patient metabolic changes following surgery. This analysis was exploratory and conducted independently of the diagnostic modeling framework. In paired models with patient-level fixed effects, no metabolite or lipoprotein feature remained significant after Benjamini-Hochberg FDR correction. Similar results were observed in prespecified technical sensitivity models incorporating batch and run-order covariates, in which no feature reached FDR significance. Thus, no FDR-robust short-term postoperative shifts in circulating metabolite or lipoprotein profiles were identified within individuals in this cohort.

## Discussion

Endometriosis remains common yet underrecognized, with delayed diagnosis continuing to limit timely management and individualized care [1]. Beyond chronic pain and impaired quality of life, it imposes a substantial socioeconomic burden and, in selected contexts, may be associated with progression to more severe disease phenotypes [7, 20, 27]. These challenges underscore the need for both clinically useful non-invasive diagnostic strategies and a better understanding of the systemic biology associated with endometriosis.

In this study, we performed a rigorous clinical evaluation of IVDr NMR-based serum metabolite and lipoprotein profiling for endometriosis across comparator settings intended to reflect both real-world diagnostic differentials and broader physiological contrasts. In the primary symptomatic-control comparison, the full IVDr NMR panel did not provide incremental diagnostic value beyond age and BMI, with consistent findings across discrimination, calibration, and decision-curve analyses. By contrast, the healthy-volunteer comparison showed substantially higher apparent separability, but this pattern was not reproduced in an independently processed temporal cohort, underscoring the importance of comparator spectrum and limited transportability when interpreting apparently strong internal performance. Beyond diagnostic evaluation, exploratory covariate-adjusted, network, and cytokine analyses suggested detectable but context-dependent systemic variation. Notably, cytokine-cytokine correlations demonstrated a reproducible within-panel immune covariance structure, whereas cross-domain cytokine-metabolite and cytokine-lipoprotein coupling was not robust after FDR correction. Short-interval longitudinal change was likewise not robust. Taken together, these findings refine expectations for serum-based diagnostic modeling in symptomatic referral populations and provide a hypothesis-generating framework for future studies using harmonized pre-analytics and clinically representative comparator spectra.

### Diagnostic evaluation and clinical relevance

#### Influence of control selection on model performance

The primary symptomatic-control comparison provides the most clinically relevant estimate of diagnostic utility because it reflects the real referral differential, in which endometriosis must be distinguished from patients with overlapping pelvic pain and gynecologic symptoms rather than from healthy physiology. In this setting, the IVDr NMR panel did not provide incremental value beyond age and BMI, indicating that apparent serum-based separation is substantially weaker in the clinically relevant differential than in broader disease-versus-health contrasts. Performance against healthy volunteers should therefore be interpreted primarily as a spectrum comparison rather than as evidence of deployable diagnostic discrimination in referral populations [28, 29]. This distinction is important because much of the biomarker literature in endometriosis has focused on disease-versus-health contrasts, whereas the translational challenge in practice is non-invasive discrimination within a symptomatic referral population [11, 30]. From a prediction-modeling perspective, these findings also emphasize that strong internal discrimination in high-dimensional omics data does not necessarily indicate clinically transferable utility [28, 31]. Comparator selection, calibration, and transportability are central to interpretation, particularly in referral populations with overlapping symptomatology [32, 33].

Interpretation of the symptomatic-control comparison also requires acknowledgment of a clinically relevant diagnostic gray zone. Although symptomatic controls lacked intraoperative evidence of endometriosis at the time of evaluation, ESHRE guidance recognizes that negative histology does not fully exclude disease, particularly when tissue confirmation is unavailable, limited, or potentially attenuated by prior hormonal therapy [11, 24, 34]. In routine care, such uncertainty can create a zone in which some patients classified as symptomatic controls may still share biological or symptom-level proximity to endometriosis, making serum-based separation intrinsically more difficult. This consideration does not alter the primary conclusion regarding limited incremental diagnostic value in the symptomatic-control setting, but it highlights the difficulty of the real-world differential diagnosis and the need for prospective validation with standardized adjudication.

At the same time, healthy-volunteer comparisons remain informative for exploratory biology because they provide a physiological reference against which broader systemic alterations can first be detected. From a translational perspective, disease-versus-health contrasts may help identify the direction of systemic metabolic disruption, whereas clinically useful biomarker development ultimately requires showing that these signals remain detectable against symptomatic comparators with overlapping clinical and biological features. In this sense, healthy-based contrasts are valuable for defining candidate biological axes, but not sufficient on their own to establish clinical utility.

#### Transportability in the independent temporal validation cohort

Independent temporal evaluation provided an additional assessment of robustness beyond the development dataset. In the clinically relevant symptomatic-control setting, discrimination was poor in the independently processed cohort. The stronger internal separation observed against healthy volunteers was likewise not reproduced. These findings indicate limited transportability of the internally derived signals under the current design and processing context. Potential contributors include temporal differences in case mix, variation in routine-care pre-analytical conditions, and comparator- or cohort-specific signal structure not fully captured by the measured technical covariates [35]. Importantly, the healthy-volunteer temporal comparison should be interpreted cautiously as an additional temporal spectrum check rather than as a substitute for clinically representative external validation [36]. In this context, healthy-volunteer contrasts are most appropriately viewed as informative for physiological and exploratory biological context, rather than as evidence of stable diagnostic transferability.

#### Technical sensitivity to batch and run order

Technical sensitivity analyses were performed to assess whether detectable batch- or run-order-related structure could plausibly explain the limited discrimination observed in the primary symptomatic-control comparison. Principal component analyses suggested low variance explained by batch and run order across most components, although one component showed a more localized technical association, indicating that technical contributions were present in part of the feature space rather than as a dominant global pattern. Moreover, incorporating batch and run-order covariates into the primary models did not materially alter performance estimates, and ComBat-based sensitivity analyses yielded similar results. Taken together, these findings suggest that although technical structure contributed some variability within the dataset, it was unlikely to be the primary explanation for the limited discrimination observed in the symptomatic-control comparison.

#### Clinical utility and prevalence-based interpretation

We further assessed potential clinical usefulness in the symptomatic-control setting using decision-curve analysis and prevalence-translated predictive values. Across clinically plausible threshold probabilities, net-benefit curves were largely similar between the baseline and full models, and PPV/NPV changed only modestly. These findings indicate that addition of the IVDr NMR panel did not meaningfully alter decision-relevant performance in the clinically relevant referral differential. This pattern is consistent with the broader interpretation of our results. Signals that separate disease from health may not be sufficiently specific to distinguish endometriosis from symptomatic presentations with overlapping complaints and potentially shared systemic perturbations. In addition, serum sampling was embedded in routine biobank workflows, and important determinants of circulating metabolite profiles, including menstrual cycle phase, time of day, fasting status, hormonal exposure, medication use, and diet, were incompletely captured, which may have further diluted disease-associated signal in the symptomatic differential [37]. Accordingly, the present findings do not support clinically meaningful incremental utility of the IVDr NMR panel under the current workflow. At the same time, the dataset remains informative as a research resource for characterizing systemic metabolic variation and for guiding future biomarker studies designed around harmonized pre-analytics and clinically representative comparator spectra.

#### Exploratory biological context

The exploratory analyses were prespecified as a parallel and independent component of the study, separate from the diagnostic modeling framework. Whereas the translational diagnostic question is most appropriately evaluated in the symptomatic-control setting, the exploratory analyses were designed to use omics data to examine broader biological patterns across comparator groups, with healthy-volunteer contrasts serving as a physiological reference and symptomatic-control contrasts serving as a clinically relevant boundary test. In this framework, the observed serum signal was more consistent with structured but context-dependent systemic variation, including patterns shared with symptomatic presentations, than with a single robust disease-specific signature that remained stable across comparator settings. At the same time, the lipoprotein-related differences appeared more restricted than the amino-acid-related changes, suggesting a potentially more endometriosis-associated pattern that warrants targeted replication under clinically representative and analytically harmonized conditions.

#### Covariate-adjusted group differences

Covariate-adjusted cross-sectional comparisons were used to provide hypothesis-generating biological context independent of diagnostic model development. The most informative differences were concentrated in contrasts involving healthy volunteers, where endometriosis cases showed lower branched-chain amino acids (valine, leucine, and isoleucine), together with lower phenylalanine, tyrosine, alanine, and lactic acid, as well as differences in creatinine and formic acid. Taken together, these findings suggest a broader serum pattern spanning amino-acid metabolism, energy-related metabolites, and lipoprotein measures, rather than isolated single-feature changes. This pattern is biologically plausible, as branched-chain amino acids are closely linked to protein turnover and immune-metabolic responses, and related shifts have been described in inflammatory and stress-associated states [38–40]. At the same time, such changes are not inherently disease-specific in serum and should therefore be interpreted cautiously.

Several of the same amino-acid differences were also observed in symptomatic controls relative to healthy volunteers. This overlap is informative because it suggests that symptomatic controls share part of the same systemic metabolic background seen in endometriosis, consistent with overlapping pelvic-pain physiology and with potentially shared inflammatory or stress-related states [41]. Previous research by Tempest et al. reported that NMR-based analysis could not identify distinguishable differences in the serum metabolome between women with and without endometriosis [42], whereas Murgia et al. identified amino acid alterations in serum using ^1^H-NMR [43]. The complexity and inconsistency of findings in this field may, at least in part, be explained by our observation that broad amino-acid-related variation is not unique to endometriosis and may therefore be diluted as a discriminatory signal in the symptomatic referral differential.

By contrast, lipoprotein-related differences appeared more restricted. In the endometriosis-versus-healthy comparison, several LDL6 subclass measures and selected HDL-related parameters were lower in endometriosis, whereas these changes were not observed in symptomatic controls versus healthy volunteers. One possible biological explanation is supported by Gibran et al. [44], who reported increased mRNA expression of the low-density lipoprotein receptor (LDLR) in lesions of deep bowel endometriosis, suggesting enhanced LDL uptake within endometriotic foci and potentially contributing to reduced circulating LDL levels and increased clearance of apolipoprotein B-100-containing particles. Because IVDr NMR lipoprotein outputs are intrinsically correlated, these findings are best interpreted as coordinated variation in lipid transport rather than as isolated single-feature effects. Such variation is biologically plausible in the context of systemic inflammation and altered metabolic homeostasis. Notably, these differences were observed in the endometriosis-versus-healthy comparison but not in the symptomatic-controls-versus-healthy comparison, suggesting that this axis may be more closely related to endometriosis than the broader amino-acid pattern. In line with the potential relevance of lipoprotein biology to disease mechanisms and translation, Bedin et al. further showed that radiolabeled LDL-like lipid nanoparticles can be taken up by endometriotic lesions via lipoprotein receptors [45], highlighting the possibility that lipoprotein-associated pathways may serve as targets for therapeutic development in endometriosis. Although this interpretation remains cautious, the lipoprotein-related signal may represent a more focused direction for targeted replication in clinically representative comparator settings.

Overall, the covariate-adjusted findings are best interpreted as context-dependent and hypothesis-generating. Healthy-volunteer contrasts captured the broadest detectable biological differences and were therefore informative for mapping systemic metabolic perturbation, whereas symptomatic-control contrasts clarified how much of this signal overlapped with clinically similar, non-endometriosis presentations. Together, these comparisons suggest that broad metabolic shifts can be detected at the disease-versus-health level, but that much of this variation is shared with symptomatic states and therefore loses discriminatory value in the referral differential. Within this broader overlap, the lipoprotein-related signal appeared more restricted, but remained modest and was not sufficient to produce FDR-robust separation in the symptomatic-control comparison. Even so, it may represent a more informative direction for targeted translational follow-up.

#### Systems-level context from WGCNA

WGCNA provided complementary systems-level context for the cross-sectional findings. After missingness filtering and redundancy pruning, the signed network identified two non-grey modules with modest but statistically significant trait associations after FDR correction. The blue module eigengene was negatively associated with overall endometriosis status, whereas the turquoise module eigengene was positively associated with symptomatic-control status. The blue module was composed predominantly of lipoprotein subclass variables, whereas the turquoise module included both lipoprotein measures and several amino acids, including branched-chain amino acids. These findings suggest that the observed serum differences were not simply isolated single-feature changes, but were also reflected in coordinated patterns of co-variation across related serum variables [46].

This systems-level perspective is particularly relevant for the lipoprotein findings. Because lipoprotein-related differences were more apparent in the endometriosis-versus-healthy comparison than in the symptomatic-controls-versus-healthy comparison, the module structure provides partial convergence with the covariate-adjusted analyses at the level of a coordinated lipoprotein-enriched pattern, rather than validation of individual feature-level associations. This point should be interpreted cautiously, especially because lipoprotein subclass measures are intrinsically correlated [47]. However, the analysis was performed after redundancy pruning, and the persistence of lipoprotein-enriched module structure suggests that the observed signal is better understood as coordinated serum variation than as a collection of isolated single-feature findings. In particular, this partial convergence is consistent with the more restricted pattern of L6-related lipoprotein measures highlighted in the covariate-adjusted contrasts. Even so, the module-level signals were modest and are best interpreted as descriptive rather than mechanistic.

#### Cytokine correlations and longitudinal exploratory findings

In the cytokine-measured subset, no cytokine-metabolite or cytokine-lipoprotein associations remained significant after FDR correction. This likely reflects a combination of modest sample size, substantial multiple-testing burden, and weak or heterogeneous serum-level immune–metabolic coupling under the present conditions. By contrast, cytokine-cytokine correlations showed a reproducible within-panel immune covariance structure, suggesting that immune co-variation was detectable at the cytokine-panel level even though robust cross-domain coupling with serum metabolites and lipoproteins was not observed in this dataset. Vodolazkaia et al. also observed an unexpected pattern in the training dataset, in which proinflammatory markers, including TNF-α, IL-6, and IL-1β, were higher in controls than in patients with endometriosis, suggesting that non-endometriotic pelvic pathology in symptomatic controls may have contributed to this increase and thereby underscoring the complexity of interpreting cytokine panels in endometriosis, particularly in the context of marked overlap with symptomatic control groups [48].

In the paired pre-/post-operative subset, we did not observe FDR-robust within-patient changes in circulating metabolites or lipoprotein measures over the short postoperative interval. Pre-operative samples were collected within 1 week before surgery, and post-operative samples were obtained 2–3 weeks after surgery during a relatively stable recovery period rather than the immediate postoperative phase. Under these conditions, these serum readouts appear unlikely to serve as short-term treatment-response markers. At the same time, the absence of rapid postoperative change is compatible with the possibility that at least part of the observed serum variation reflects relatively stable systemic traits rather than immediately reversible short-term states.

### Strengths and limitations

This study has several strengths. The diagnostic analyses were anchored in surgically confirmed endometriosis and prioritized a clinically realistic comparator spectrum by using symptomatic controls for the primary comparison. Model evaluation used a prespecified, leakage-resistant framework with fully nested repeated cross-validation and considered calibration and decision-curve analysis in addition to discrimination. Robustness was further examined in an independently processed temporal cohort, including an exploratory healthy-volunteer comparison. Beyond prediction, the exploratory biological analyses were explicitly separated from diagnostic modeling and incorporated covariate-adjusted comparisons across healthy volunteers, symptomatic controls, and endometriosis cases, allowing assessment of both broad physiological contrasts and clinically relevant overlap. In this respect, the study addresses an important gap in the endometriosis biomarker literature, in which disease-versus-healthy comparisons have often been emphasized more than clinically relevant symptomatic referral differentials.

Several limitations should also be considered. The study was conducted at a single center and did not include true multicenter external validation. Accordingly, both the transportability of the diagnostic findings and their generalizability across institutions and pre-analytical workflows remain limited. Sample collection followed routine clinical practice, and important determinants of serum metabolomics, including fasting status, time of day, menstrual cycle phase, hormonal exposure, medication use, and diet, were incompletely captured and could not be systematically adjusted. The cytokine analyses were restricted to a subset of participants, limiting sensitivity for cross-domain immune–metabolic analyses under a substantial multiple-testing burden. The IVDr NMR feature set is platform-specific and highly structured, and residual unmeasured confounding may remain despite covariate adjustment and technical sensitivity analyses. In addition, the healthy-volunteer temporal comparison was exploratory and should not be interpreted as equivalent to a clinically representative external validation benchmark.

## Conclusions

In this study, the IVDr NMR serum metabolite-lipoprotein panel showed strong apparent discrimination when endometriosis was compared with healthy volunteers, but this did not translate to the clinically relevant symptomatic-control setting, where the panel did not provide consistent incremental value beyond age and BMI. These findings highlight the importance of comparator spectrum and indicate that disease-versus-health separation should not be assumed to reflect clinically transferable diagnostic utility in referral populations.

An additional age/BMI-matched sensitivity analysis further suggested that the strong apparent discrimination against healthy volunteers was partly influenced by demographic (age) imbalance, but a residual metabolite-lipoprotein signal remained detectable after matching. This residual signal does not alter the overall conclusion regarding limited clinical diagnostic utility in the symptomatic-control setting, but remains biologically informative and relevant for future research.

At the same time, the exploratory analyses identified biologically informative patterns at the serum level. Healthy-based contrasts revealed a broader systemic metabolic signal involving branched-chain and related amino acids, alanine, lactic acid, and coordinated lipoprotein-related differences. Part of the amino-acid signal was also present in symptomatic controls, consistent with shared symptom-state physiology, whereas the lipoprotein-related changes appeared more restricted and were supported at a systems level by lipoprotein-enriched network structure. In addition, cytokine-cytokine correlations revealed a reproducible within-panel immune covariance structure, although robust cross-domain immune–metabolic coupling was not observed under the present conditions. Together, these findings suggest that serum metabolomics may be more informative for defining biological axes of variation than for immediate diagnostic deployment under the present conditions, and that lipoprotein-related measures may represent a more focused direction for targeted follow-up.

Future studies should prespecify the intended clinical use case and prioritize multicenter recruitment with harmonized pre-analytical procedures and clinically representative symptomatic comparators. More complete phenotyping of key determinants of serum metabolomics, including menstrual cycle phase, hormonal exposure, fasting status, time of day, medication use, and symptom trajectories, will be essential to distinguish shared symptom-state physiology from more disease-associated variation. Longer-term longitudinal studies will be needed to determine whether lipoprotein-related patterns reflect relatively stable systemic traits, track symptom-associated states, or relate more directly to lesion biology. As the present study was limited to circulation-level profiling, future work should, where feasible, integrate serum analyses with tissue-adjacent sampling and spatially resolved approaches, including Matrix-assisted laser desorption/ionization (MALDI) based analyses, to clarify how systemic and local metabolic alterations are linked.

## Supplementary Information

Below is the link to the electronic supplementary material.

Supplementary Material 1: Table S1. Table S1 – Annotation of the abbreviations used by the IVDr lipoprotein profiling analysis.

Supplementary Material 2: Tables S2-S3. Table S2 – Assay-specific lower limits of quantitation (LLOQ, pg/mL) used to define missing cytokine values. Table S3 – Membership of non-grey WGCNA modules identified in the signed network analysis.

Supplementary Material 3: Figure S1-S5. Figure S1 – Age and BMI density distributions across development, temporal validation, and matched comparison panels. Figure S2 – Principal component analysis of the D1 feature space colored by technical and clinical factors. Figure S3 – Covariate-adjusted effect sizes for FDR-significant healthy-based contrasts. Supplementary Figure S4 – Complete three-group raincloud plots for all 16 unique features significant in at least one covariate-adjusted mechanistic contrast. Figure S5 – Sample clustering and soft-threshold selection for Weighted Gene Co-expression Network Analysis (WGCNA).

Supplementary Material 4: Table S4A. Table S4A – Exploratory nominal cytokine-metabolite and cytokine-lipoprotein associations (Spearman raw *p* < 0.05) in the endometriosis-only and sensitivity cohorts.

Supplementary Material 5: Table S4B. Table S4B – FDR-significant cytokine-cytokine correlations in the endometriosis-only and sensitivity cohorts.

## Data Availability

A manuscript-focused reproducibility package for the analyses reported in this study is publicly available at Zenodo (DOI: 10.5281/zenodo.19185350) [49]. The public deposit includes selected analysis scripts, environment metadata, execution notes, fixed random seeds, and summary-level frozen analytical artefacts supporting the reported workflow. Individual-level data are not publicly available because this study combines human participant samples and metadata obtained under multiple cohort-specific ethics approvals, biobank governance frameworks, third-party transfer agreements, and ongoing-study restrictions. De-identified summary-level results underlying the main figures and tables are included in the Zenodo package or may be obtained from the corresponding authors upon reasonable request. Requests for controlled access to participant-level data remain subject to institutional approval, applicable ethics requirements, and material/data transfer agreements.

## Abbreviations

^1^H-NMR: Proton Nuclear Magnetic Resonance spectroscopy
ART: Assisted reproductive technologies
ESHRE: European Society of Human Reproduction and Embryology
WGCNA: Weighted Gene Co-expression Network Analysis
AUC: Area under the receiver operating characteristic curve
IVDr: In Vitro Diagnostics Research
UKT: University Hospital Tübingen
CiC BioGUNE: Center for Cooperative Research in Biosciences
EMT: Endometriosis
SC: Symptomatic Controls
HV: Healthy Volunteers
BMI: Body Mass Index
GDPR: the European General Data Protection Regulation
LLOQ: Lower Limit of Quantitation
TSP: Trimethylsilylpropanoic acid
D1: Primary diagnostic comparison
D2: Secondary diagnostic comparison
SMD: Standardized mean difference
QC: Quality control
IL: Interleukin
IFN: Interferon
TNF: Tumor necrosis factor
MCP-1: Monocyte chemoattractant protein-1
HDL: High-density lipoprotein
LDL: Low-density lipoprotein
VLDL: Very-low-density lipoprotein
LDLR: Low-density lipoprotein receptor
MRI: Magnetic resonance imaging
ABA1 or AB/A1: Apolipoprotein-B100/Apolipoprotein-A1
H1A1: Apolipoprotein-A1 HDL-1
H1A2: Apolipoprotein-A2 HDL-1
H1CH: Cholesterol HDL-1
H1FC: Free Cholesterol HDL-1
H1PL: Phospholipids HDL-1
H1TG: Triglycerides HDL-1
H2A1: Apolipoprotein-A1 HDL-2
H2A2: Apolipoprotein-A2 HDL-2
H2CH: Cholesterol HDL-2
H2FC: Free Cholesterol HDL-2
H2PL: Phospholipids HDL-2
H2TG: Triglycerides HDL-2
H3A1: Apolipoprotein-A1 HDL-3
H3A2: Apolipoprotein-A2 HDL-3
H3CH: Cholesterol HDL-3
H3FC: Free Cholesterol HDL-3
H3PL: Phospholipids HDL-3
H3TG: Triglycerides HDL-3
H4A1: Apolipoprotein-A1 HDL-4
H4A2: Apolipoprotein-A2 HDL-4
H4CH: Cholesterol HDL-4
H4FC: Free Cholesterol HDL-4
H4PL: Phospholipids HDL-4
H4TG: Triglycerides HDL-4
HDA1: HDL-Apolipoprotein-A1
HDA2: HDL-Apolipoprotein-A2
HDCH: HDL-Cholesterol
HDFC: HDL Free Cholesterol
HDPL: HDL Phospholipids
HDTG: HDL Triglycerides
IDAB: IDL-Apolipoprotein-B100
IDCH: IDL Cholesterol
IDFC: IDL Free Cholesterol
IDPL: IDL Phospholipids
IDPN: ILDL Particle Number
IDTG: IDL Triglycerides
L1AB: Apolipoprotein-B100 LDL-1
L1CH: Cholesterol LDL-1
L1FC: Free Cholesterol LDL-1
L1PL: Phospholipids LDL-1
L1PN: Particle Number LDL-1
L1TG: Triglycerides LDL-1
L2AB: Apolipoprotein-B100 LDL-2
L2CH: Cholesterol LDL-2
L2FC: Free Cholesterol LDL-2
L2PL: Phospholipids LDL-2
L2PN: Particle Number LDL-2
L2TG: Triglycerides LDL-2
L3AB: Apolipoprotein-B100 LDL-3
L3CH: Cholesterol LDL-3
L3FC: Free Cholesterol LDL-3
L3PL: Phospholipids LDL-3
L3PN: Particle Number LDL-3
L3TG: Triglycerides LDL-3
L4AB: Apolipoprotein-B100 LDL-4
L4CH: Cholesterol LDL-4
L4FC: Free Cholesterol LDL-4
L4PL: Phospholipids LDL-4
L4PN: Particle Number LDL-4
L4TG: Triglycerides LDL-4
L5AB: Apolipoprotein-B100 LDL-5
L5CH: Cholesterol LDL-5
L5FC: Free Cholesterol LDL-5
L5PL: Phospholipids LDL-5
L5PN: Particle Number LDL-5
L5TG: Triglycerides LDL-5
L6AB: Apolipoprotein-B100 LDL-6
L6CH: Cholesterol LDL-6
L6FC: Free Cholesterol LDL-6
L6PL: Phospholipids LDL-6
L6PN: Particle Number LDL-6
L6TG: Triglycerides LDL-6
LDAB: LDL-Apolipoprotein-B100
LDCH: LDL-Cholesterol
LDFC: LDL Free Cholesterol
LDHD or LD/HD: LDL-cholesterol/HDL-cholesterol
LDPL: LDL Phospholipids
LDPN: LDL Particle Number
LDTG: LDL Triglycerides
TBPN: Total Particle Number (apolipoprotein-B100 carrying particles)
TPA1: Total Plasma Apolipoprotein-A1
TPA2: Total Plasma Apolipoprotein-A2
TPAB: Total Plasma Apolipoprotein-B100
TPCH: Total Plasma Cholesterol
TPTG: Total Plasma Triglycerides
V1CH: Cholesterol VLDL-1
V1FC: Free Cholesterol VLDL-1
V1PL: Phospholipids VLDL-1
V1TG: Triglycerides VLDL-1
V2CH: Cholesterol VLDL-2
V2FC: Free Cholesterol VLDL-2
V2PL: Phospholipids VLDL-2
V2TG: Triglycerides VLDL-2
V3CH: Cholesterol VLDL-3
V3FC: Free Cholesterol VLDL-3
V3PL: Phospholipids VLDL-3
V3TG: Triglycerides VLDL-3
V4CH: Cholesterol VLDL-4
V4FC: Free Cholesterol VLDL-4
V4PL: Phospholipids VLDL-4
V4TG: Triglycerides VLDL-4
V5CH: Cholesterol VLDL-5
V5FC: Free Cholesterol VLDL-5
V5PL: Phospholipids VLDL-5
V5TG: Triglycerides VLDL-5
VLAB: VLDL-Apolipoprotein-B100
VLCH: VLDL Cholesterol
VLFC: VLDL Free Cholesterol
VLPL: VLDL Phospholipids
VLPN: VLDL Particle Number
VLTG: VLDL Triglycerides
NDIE: Non-Deep Infiltrating Endometriosis
DIE: Deep Infiltrating Endometriosis
PC: Principal component
FDR: False Discovery Rate
PPV: Positive Predictive Value
NPV: Negative Predictive Value
NRI: Net reclassification improvement
CI: Confidence Interval
IDI: Integrated discrimination improvement
BMBF: German Federal Ministry of Education and Research
MALDI: Matrix-assisted laser desorption/ionization
