# Supplementary material for: NMR-based serum metabolite and lipoprotein profiling for endometriosis across clinically relevant and physiological comparator settings: assessment of diagnostic utility and exploratory biological signals
Source: BMC Med. 2026 Jun 17;24:362. doi: 10.1186/s12916-026-04999-2 (PMC13277188; doi:10.1186/s12916-026-04999-2)
Supplement: Supplementary file 1 — Supplementary Material 1: Table S1. Table S1 – Annotation of the abbreviations used by the IVDr lipoprotein profiling analysis. [file 12916_2026_4999_MOESM1_ESM.docx]

**Supplementary Table S2.** Assay-specific lower limits of quantitation (LLOQ, pg/mL) used to define missing cytokine values.

| **Cytokine** | **LLOQ, pg/mL** |
| --- | --- |
| IL-1β | 1.5 ± 0.6 |
| IFN-α2 | 2.1 ± 0.2 |
| IFN-γ | 1.3 ± 1.0 |
| TNF-α | 0.9 ± 0.8 |
| MCP-1 | 1.1 ± 1.2 |
| IL-6 | 1.5 ± 0.7 |
| IL-8 | 2.0 ± 0.5 |
| IL-10 | 2.0 ± 0.5 |
| IL-12p70 | 2.0 ± 0.2 |
| IL-17A | 0.5 ± 0.0 |
| IL-18 | 2.0 ± 0.5 |
| IL-23 | 1.8 ± 0.1 |
| IL-33 | 4.4 ± 1.5 |

**Supplementary Table S3.** Membership of non-grey WGCNA modules identified in the signed network analysis.

| **Module** | **Number of features** | **Features** |
| --- | --- | --- |
| Blue | 26 | TPCH, LDHD, ABA1, TBPN, L3PN, L4PN, LDTG, LDFC, L1TG, L2TG, L3TG, L4TG, L5TG, L6TG, L3CH, L4CH, L5CH, L6CH, L3FC, L4FC, L5FC, L6FC, L1PL, L4PL, L5PL, L2AB |
| Turquoise | 31 | IDPN, VLTG, IDTG, VLCH, IDCH, IDPL, V1TG, V2TG, V3TG, V4TG, V5TG, V1CH, V2CH, V3CH, V4CH, V5CH, V1FC, V2FC, V3FC, V4FC, V5FC, V1PL, V3PL, V5PL, H2TG, H3TG, H4TG, Isoleucine, Leucine, Threonine, Valine |
