# Supplementary material for: NMR-based serum metabolite and lipoprotein profiling for endometriosis across clinically relevant and physiological comparator settings: assessment of diagnostic utility and exploratory biological signals
Source: BMC Med. 2026 Jun 17;24:362. doi: 10.1186/s12916-026-04999-2 (PMC13277188; doi:10.1186/s12916-026-04999-2)
Supplement: Supplementary file 4 — Supplementary Material 4: Table S4A. Table S4A – Exploratory nominal cytokine-metabolite and cytokine-lipoprotein associations (Spearman raw p < 0.05) in the endometriosis-only and sensitivity cohorts. [file 12916_2026_4999_MOESM4_ESM.docx]

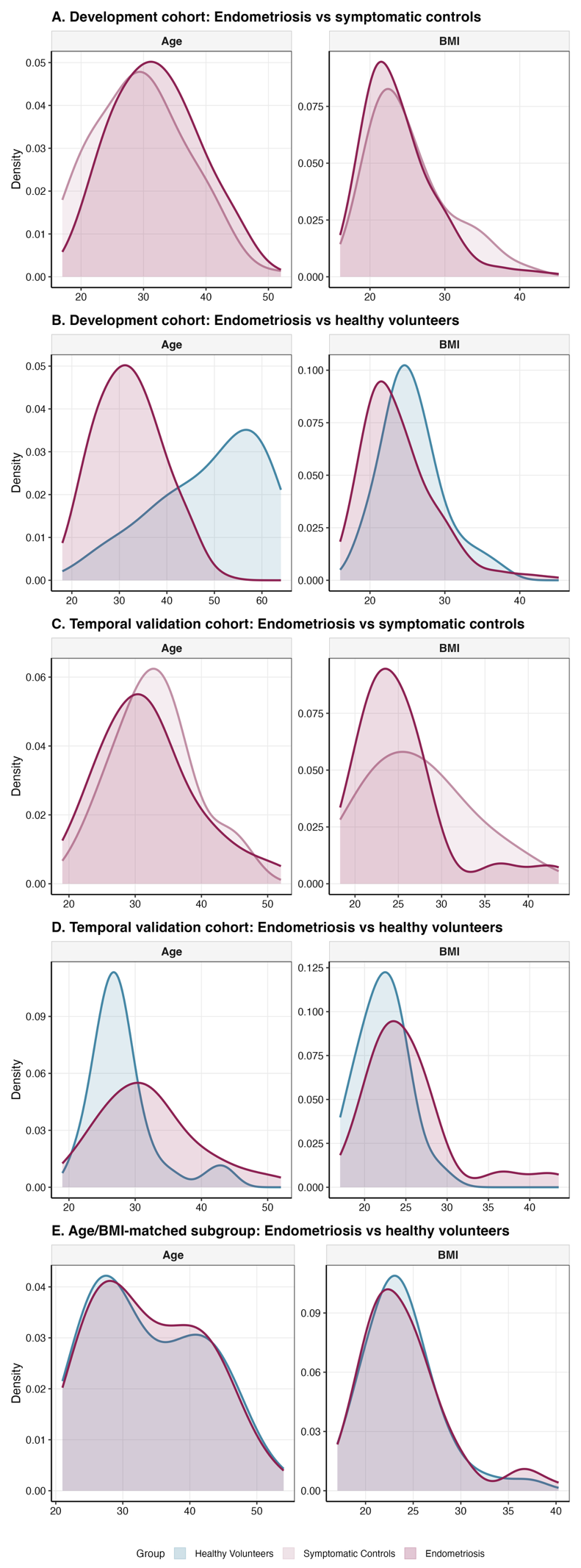


***Supplementary Figure S1.*** *Age and BMI density distributions across development, temporal validation, and matched comparison panels.*

*Kernel density plots are shown for age and body mass index (BMI) in the development cohort and the independent temporal validation cohort for the primary comparison (endometriosis vs symptomatic controls) and the secondary comparison (endometriosis vs healthy volunteers). The plots show greater age and, to a lesser extent, BMI separation in comparisons involving healthy volunteers, whereas endometriosis and symptomatic controls showed more overlapping demographic distributions. Panel E shows an additional age/BMI-balanced subgroup for the endometriosis-versus-healthy comparison derived by propensity score matching, in which post-matching density plots indicate improved covariate balance. Together, these plots provide context for the baseline age + BMI model and support interpretation of comparator-spectrum differences across analyses.*


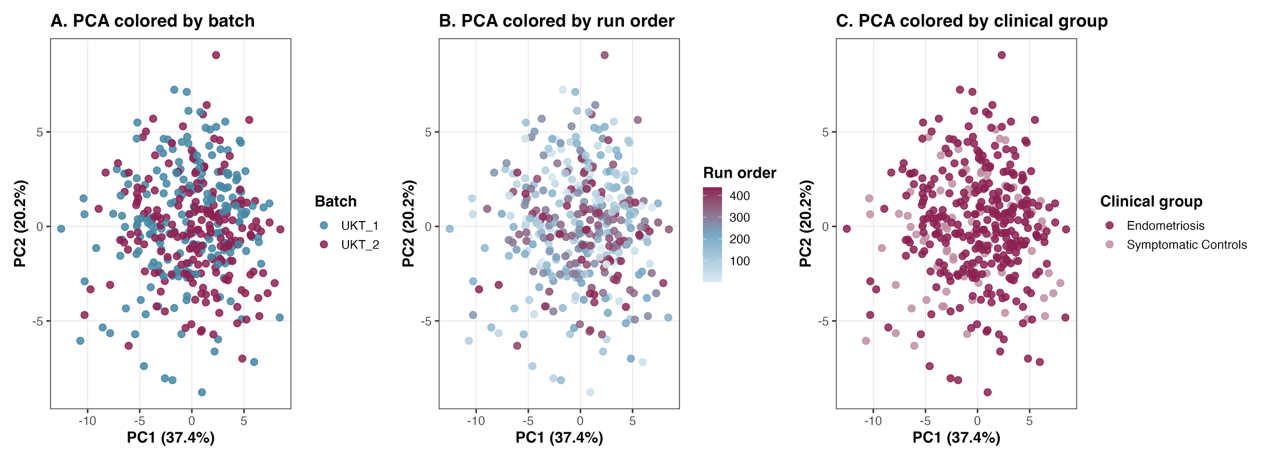


***Supplementary Figure S2.*** *Principal component analysis of the D1 feature space colored by technical and clinical factors.*

*PCA score plots for the primary comparison panel (endometriosis vs symptomatic controls) are shown with points colored by batch (A), analytical run order (B), and clinical group (C). Axes indicate the proportion of variance explained by PC1 and PC2. Overall, the plots show substantial overlap across batches, run order, and clinical groups, consistent with the absence of strong global clustering by either technical or clinical factors in the first two principal components. These visual patterns support the interpretation that detectable technical structure, where present, was modest and localized rather than dominant at the level of the major variance components.*


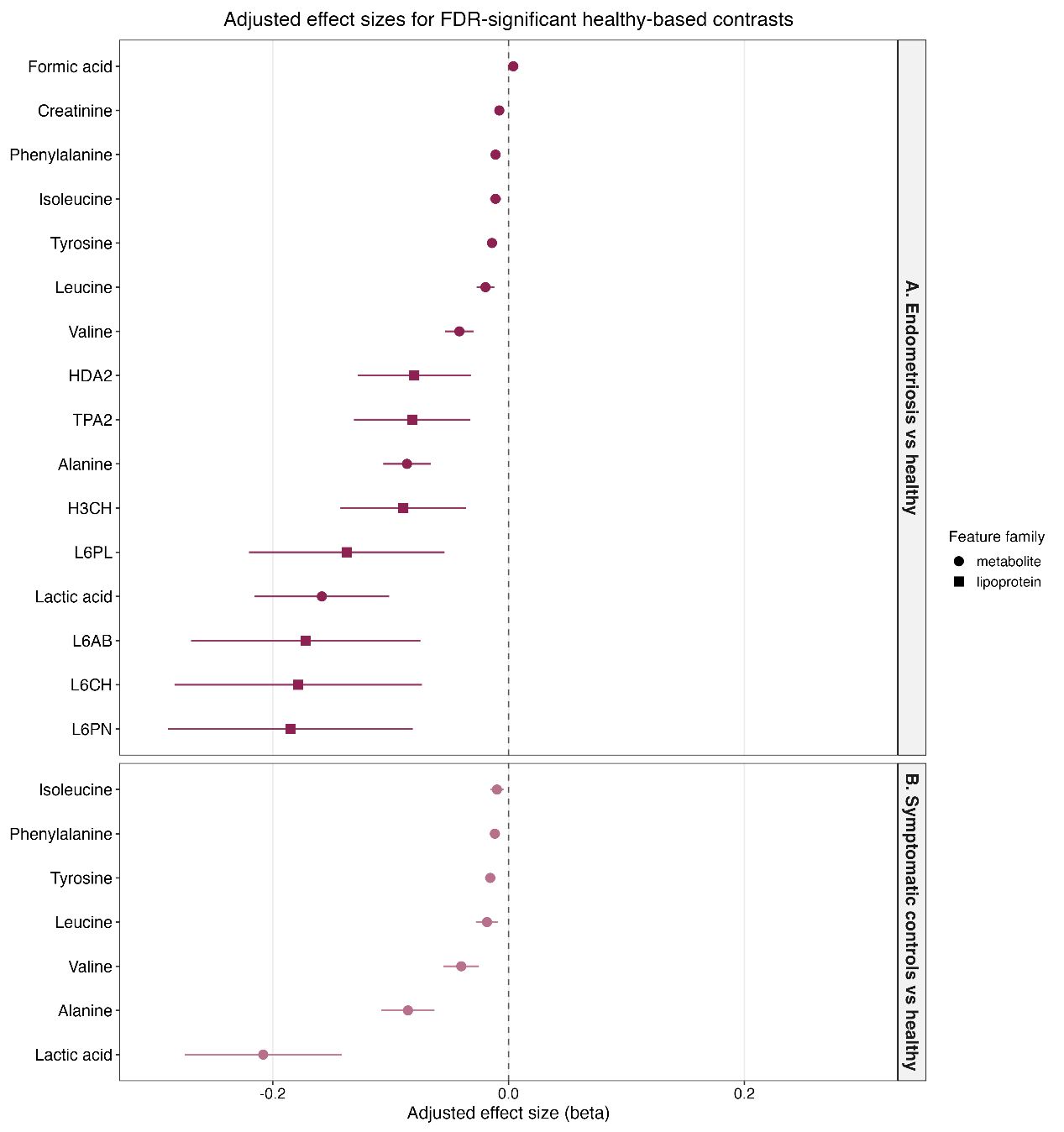


***Supplementary Figure S3.*** *Covariate-adjusted effect sizes for FDR-significant healthy-based contrasts.*

***A*** *Endometriosis versus healthy volunteers.* ***B*** *Symptomatic controls versus healthy volunteers. Points represent adjusted regression coefficients (beta) from feature-wise covariate-adjusted linear models, and horizontal lines indicate 95% confidence intervals. Circles denote metabolites and squares denote lipoprotein features. Negative effect estimates indicate lower feature levels relative to healthy volunteers. The figure shows that several amino-acid and lactic-acid differences were shared across both healthy-based contrasts, whereas lipoprotein-related differences were observed predominantly in the endometriosis-versus-healthy comparison.*


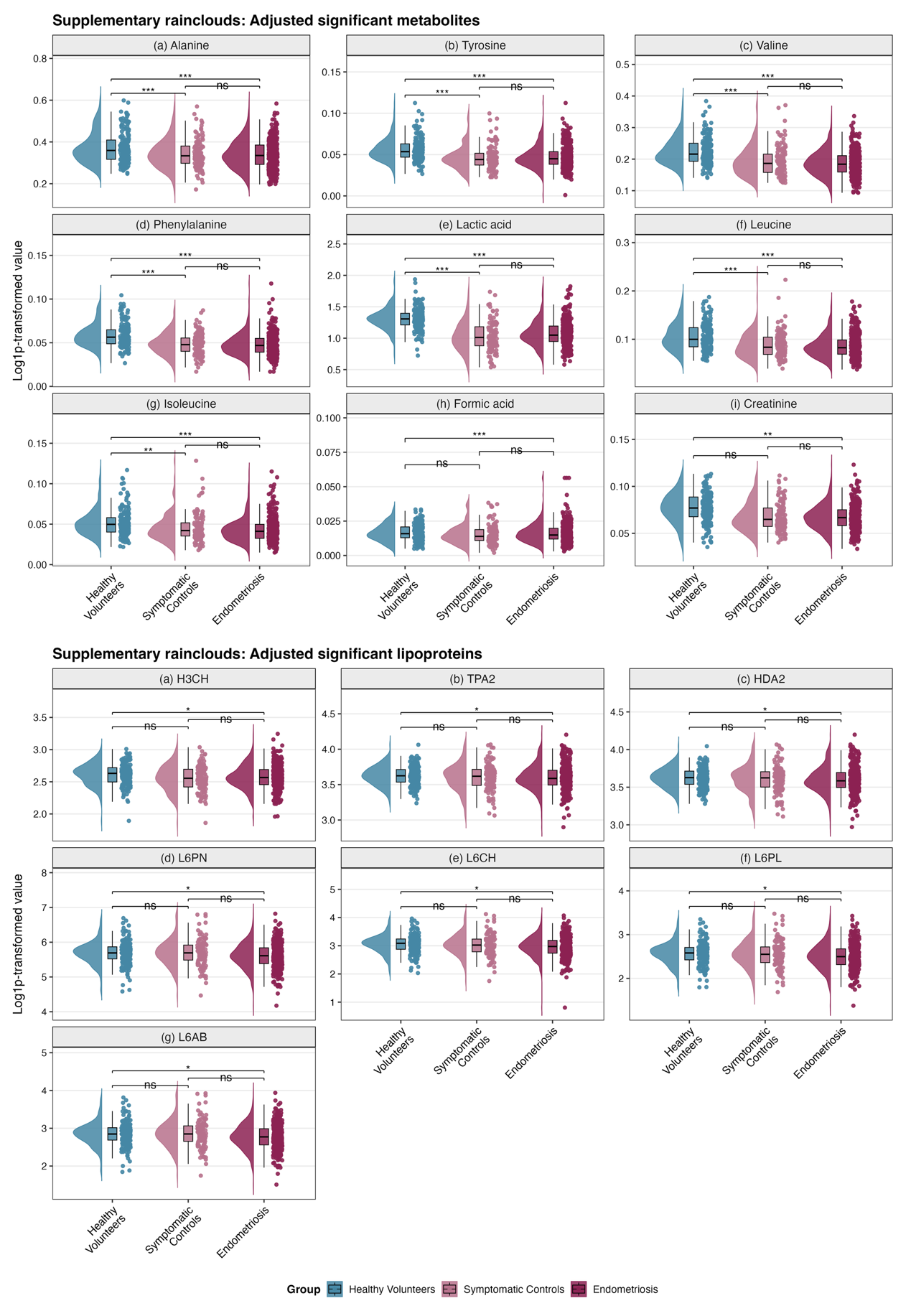


***Supplementary Figure S4.*** *Complete three-group raincloud plots for all 16 unique features significant in at least one covariate-adjusted mechanistic contrast.*

*Raincloud plots show the distributions of log1p-transformed values for healthy volunteers, symptomatic controls, and endometriosis cases. The displayed features include all 16 unique metabolites and lipoprotein measures that were significant in at least one covariate-adjusted cross-sectional comparison after FDR correction. Boxplots summarize the median and interquartile range, and points indicate individual samples. Statistical annotations represent pairwise comparisons derived from the covariate-adjusted models; significance symbols correspond to FDR-adjusted q values (*FDR < 0.05, **FDR < 0.01, ***FDR < 0.001; ns, not significant). Overall, the plots illustrate that amino-acid-related differences were more broadly shared across healthy-based contrasts, whereas lipoprotein-related differences appeared more restricted.*


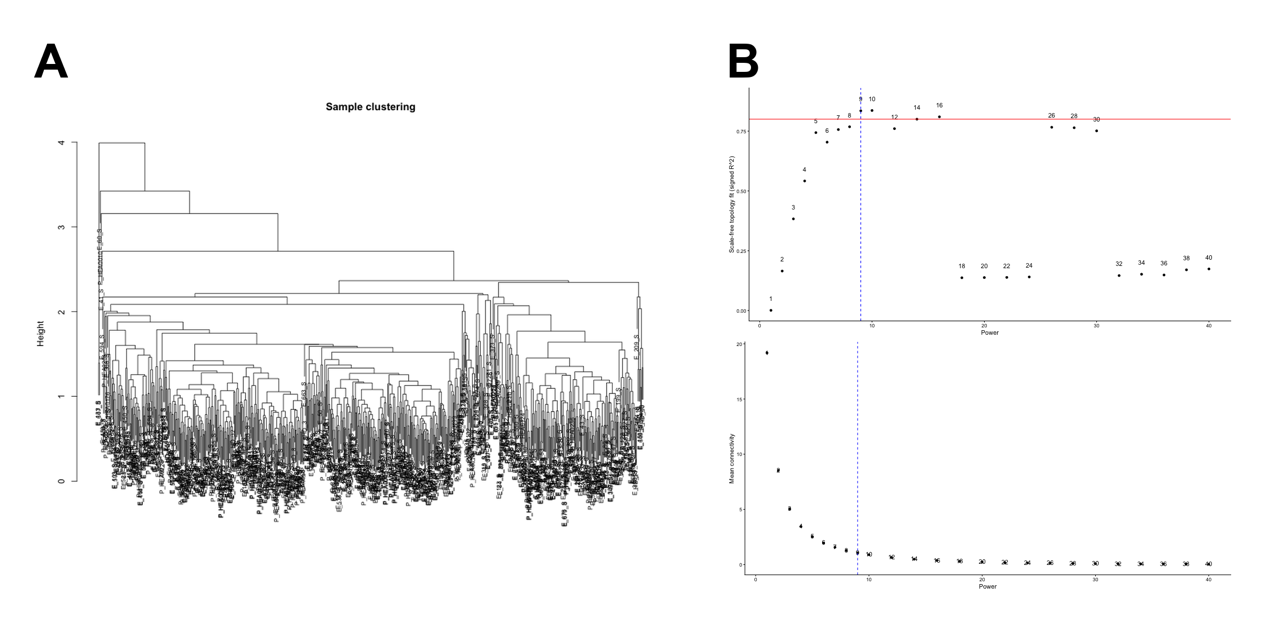


***Supplementary Figure S5.*** *Sample clustering and soft-threshold selection for Weighted Gene Co-expression Network Analysis (WGCNA)*

***A*** *Hierarchical clustering dendrogram of samples used for WGCNA, performed to assess overall sample structure and screen for potential outliers prior to network construction. No dominant outlier cluster prompting sample exclusion was identified.*

***B*** *Soft-threshold selection plots for the signed WGCNA network. The upper panel shows the scale-free topology fit index across candidate soft-thresholding powers, and the lower panel shows the corresponding mean connectivity. The dashed vertical line marks the selected soft-thresholding power (β = 9), chosen to balance approximate scale-free topology with preservation of network connectivity for downstream module detection.*
