# Supplementary material for: NMR-based serum metabolite and lipoprotein profiling for endometriosis across clinically relevant and physiological comparator settings: assessment of diagnostic utility and exploratory biological signals
Source: BMC Med. 2026 Jun 17;24:362. doi: 10.1186/s12916-026-04999-2 (PMC13277188; doi:10.1186/s12916-026-04999-2)

Table S1. Abbreviations used within Bruker’s IVDr lipoprotein profiling analysis.

Number

Parameter

Full name

Unit

1

2

3

4

5

6

7

8

9

0

1

2

3

4

5

6

7

8

9

0

1

2

3

4

5

6

7

8

9

0

1

2

3

4

5

6

7

8

9

0

1

2

ABA1

H1A1

H1A2

H1CH

H1FC

H1PL

H1TG

H2A1

H2A2

H2CH

H2FC

H2PL

H2TG

H3A1

H3A2

H3CH

H3FC

H3PL

H3TG

H4A1

H4A2

H4CH

H4FC

H4PL

H4TG

HDA1

HDA2

HDCH

HDFC

HDPL

HDTG

IDAB

IDCH

IDFC

Apolipoprotein‐B100/Apolipoprotein‐A1

Apolipoprotein‐A1 HDL‐1

Apolipoprotein‐A2 HDL‐1

Cholesterol HDL‐1

‐

mg/dL

mg/dL

mg/dL

mg/dL

mg/dL

mg/dL

mg/dL

mg/dL

mg/dL

mg/dL

mg/dL

mg/dL

mg/dL

mg/dL

mg/dL

mg/dL

mg/dL

mg/dL

mg/dL

mg/dL

mg/dL

mg/dL

mg/dL

mg/dL

mg/dL

mg/dL

mg/dL

mg/dL

mg/dL

mg/dL

mg/dL

mg/dL

mg/dL

mg/dL

nmol/L

mg/dL

mg/dL

mg/dL

mg/dL

mg/dL

nmol/L

Free Cholesterol HDL‐1

Phospholipids HDL‐1

Triglycerides HDL‐1

Apolipoprotein‐A1 HDL‐2

Apolipoprotein‐A2 HDL‐2

Cholesterol HDL‐2

Free Cholesterol HDL‐2

Phospholipids HDL‐2

Triglycerides HDL‐2

Apolipoprotein‐A1 HDL‐3

Apolipoprotein‐A2 HDL‐3

Cholesterol HDL‐3

Free Cholesterol HDL‐3

Phospholipids HDL‐3

Triglycerides HDL‐3

Apolipoprotein‐A1 HDL‐4

Apolipoprotein‐A2 HDL‐4

Cholesterol HDL‐4

Free Cholesterol HDL‐4

Phospholipids HDL‐4

Triglycerides HDL‐4

HDL‐Apolipoprotein‐A1

HDL‐Apolipoprotein‐A2

HDL‐Cholesterol

HDL Free Cholesterol

HDL Phospholipids

HDL Triglycerides

IDL‐Apolipoprotein‐B100

IDL Cholesterol

IDL Free Cholesterol

IDL Phospholipids

ILDL Particle Number

IDL Triglycerides

Apolipoprotein‐B100 LDL‐1

Cholesterol LDL‐1

Free Cholesterol LDL‐1

Phospholipids LDL‐1

Particle Number LDL‐1

1

1

1

1

1

1

1

1

1

1

2

2

2

2

2

2

2

2

2

2

3

3

3

3

3

3

3

3

3

3

4

4

4

IDPL

IDPN

IDTG

L1AB

L1CH

L1FC

L1PL

L1PN


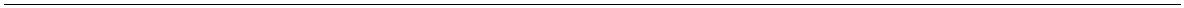

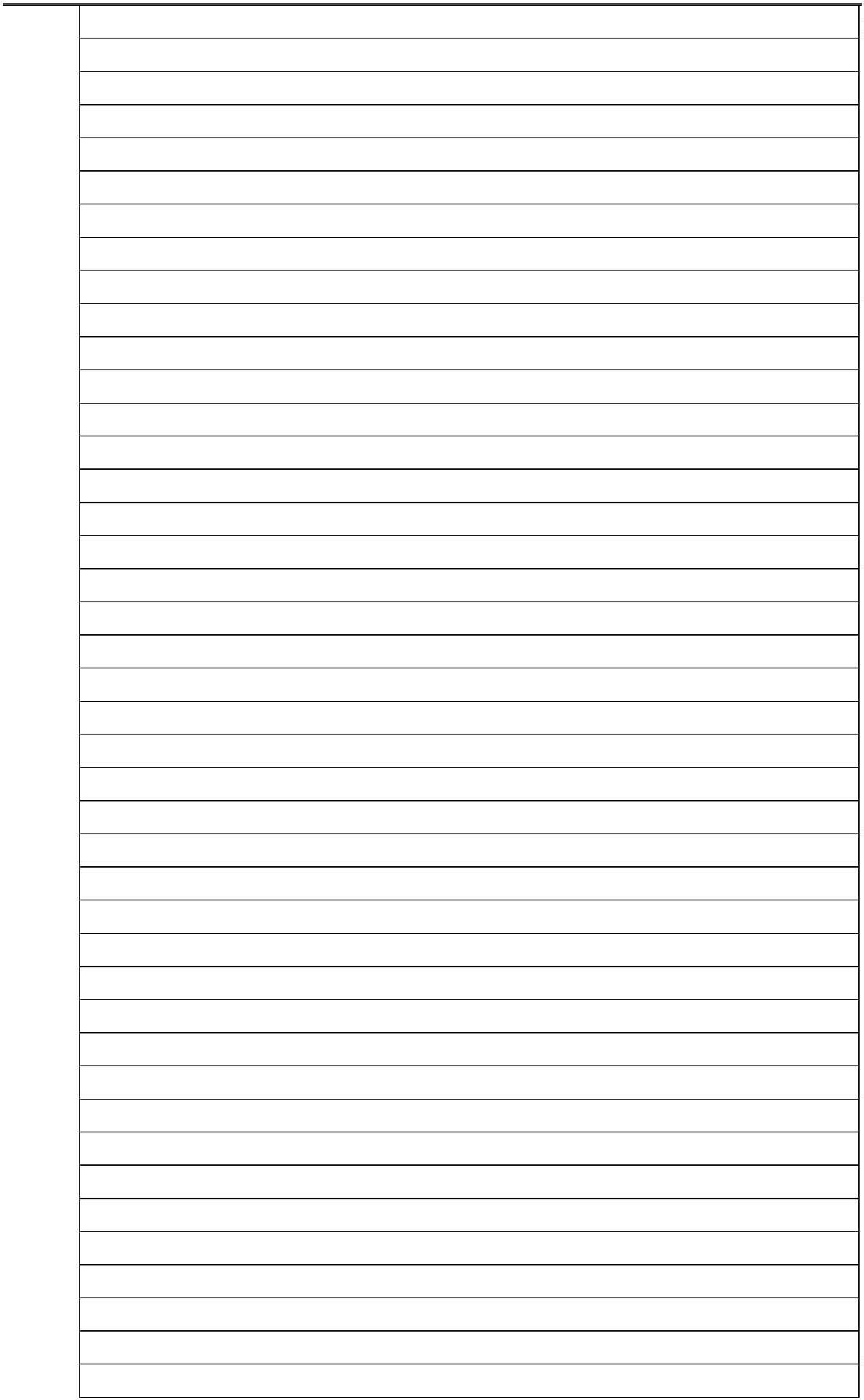


4

4

4

4

4

4

4

5

5

5

5

5

5

5

5

5

5

6

6

6

6

6

6

6

6

6

6

7

7

7

7

7

7

7

7

7

7

8

8

3

4

5

6

7

8

9

0

1

2

3

4

5

6

7

8

9

0

1

2

3

4

5

6

7

8

9

0

1

2

3

4

5

6

7

8

9

0

1

L1TG

L2AB

L2CH

L2FC

L2PL

L2PN

L2TG

L3AB

L3CH

L3FC

L3PL

L3PN

L3TG

L4AB

L4CH

L4FC

L4PL

L4PN

L4TG

L5AB

L5CH

L5FC

L5PL

L5PN

L5TG

L6AB

L6CH

L6FC

L6PL

L6PN

L6TG

LDAB

LDCH

LDFC

LDHD

LDPL

LDPN

LDTG

TBPN

Triglycerides LDL‐1

Apolipoprotein‐B100 LDL‐2

Cholesterol LDL‐2

Free Cholesterol LDL‐2

Phospholipids LDL‐2

Particle Number LDL‐2

Triglycerides LDL‐2

Apolipoprotein‐B100 LDL‐3

Cholesterol LDL‐3

Free Cholesterol LDL‐3

Phospholipids LDL‐3

Particle Number LDL‐3

Triglycerides LDL‐3

Apolipoprotein‐B100 LDL‐4

Cholesterol LDL‐4

Free Cholesterol LDL‐4

Phospholipids LDL‐4

Particle Number LDL‐4

Triglycerides LDL‐4

Apolipoprotein‐B100 LDL‐5

Cholesterol LDL‐5

Free Cholesterol LDL‐5

Phospholipids LDL‐5

Particle Number LDL‐5

Triglycerides LDL‐5

mg/dL

mg/dL

mg/dL

mg/dL

mg/dL

nmol/L

mg/dL

mg/dL

mg/dL

mg/dL

mg/dL

nmol/L

mg/dL

mg/dL

mg/dL

mg/dL

mg/dL

nmol/L

mg/dL

mg/dL

mg/dL

mg/dL

mg/dL

nmol/L

mg/dL

mg/dL

mg/dL

mg/dL

mg/dL

nmol/L

mg/dL

mg/dL

mg/dL

mg/dL

‐

Apolipoprotein‐B100 LDL‐6

Cholesterol LDL‐6

Free Cholesterol LDL‐6

Phospholipids LDL‐6

Particle Number LDL‐6

Triglycerides LDL‐6

LDL‐Apolipoprotein‐B100

LDL‐Cholesterol

LDL Free Cholesterol

LDL‐cholesterol/HDL‐cholesterol

LDL Phospholipids

mg/dL

nmol/L

mg/dL

nmol/L

LDL Particle Number

LDL Triglycerides

Total Particle Number

(apolipoprotein‐B100 carrying particles)

8

8

8

8

8

8

2

3

4

5

6

7

TPA1

TPA2

TPAB

TPCH

TPTG

V1CH

Total Plasma Apolipoprotein‐A1

mg/dL

mg/dL

mg/dL

mg/dL

mg/dL

mg/dL

Total Plasma Apolipoprotein‐A2

Total Plasma Apolipoprotein‐B100

Total Plasma Cholesterol

Total Plasma Triglycerides

Cholesterol VLDL‐1


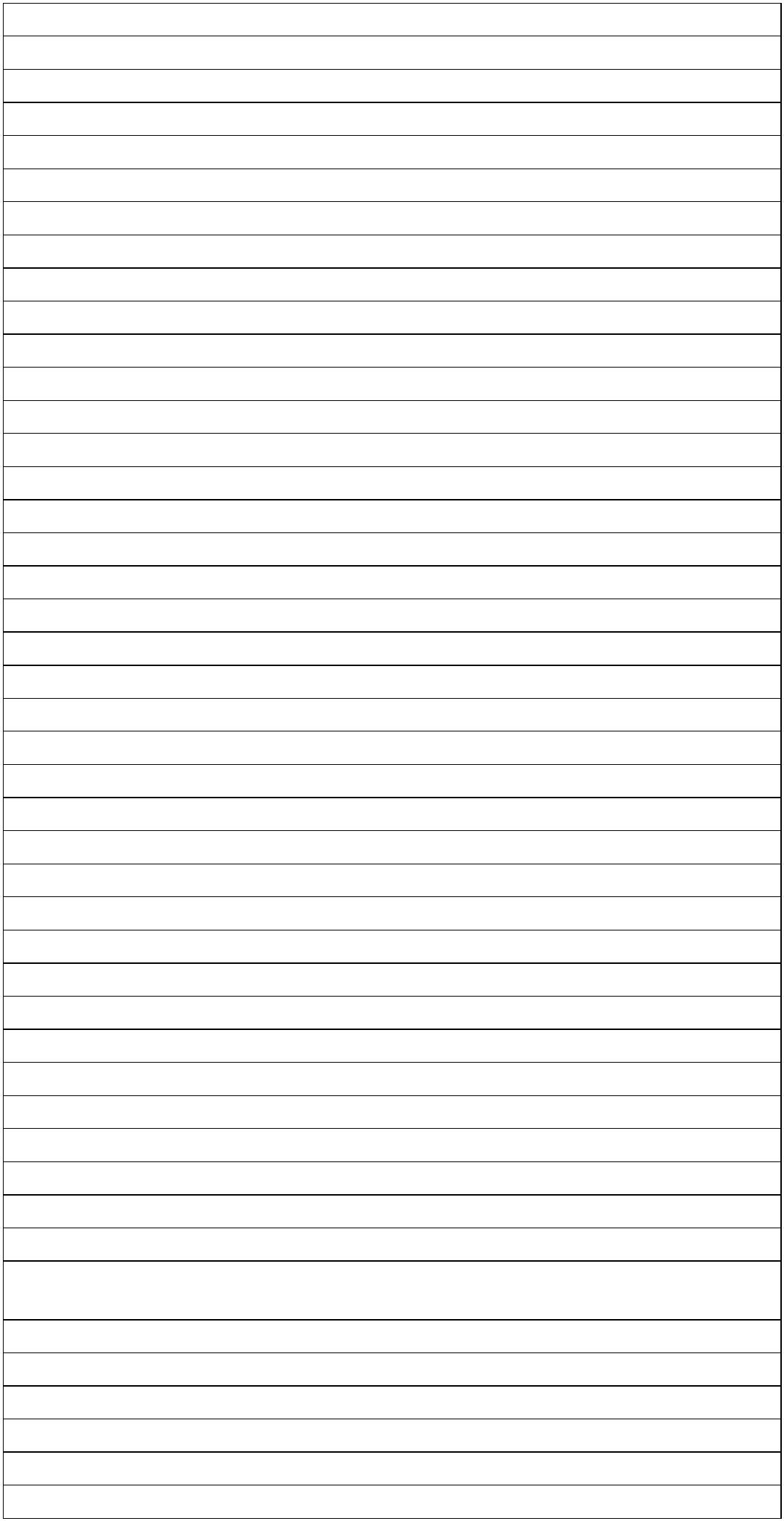


8

8

9

9

9

9

9

9

9

9

9

9

00

01

02

03

04

05

06

07

08

09

10

11

12

8

9

0

1

2

3

4

5

6

7

8

9

V1FC

V1PL

V1TG

V2CH

V2FC

V2PL

V2TG

V3CH

V3FC

V3PL

V3TG

V4CH

V4FC

V4PL

V4TG

V5CH

V5FC

V5PL

V5TG

VLAB

VLCH

VLFC

VLPL

VLPN

VLTG

Free Cholesterol VLDL‐1

Phospholipids VLDL‐1

Triglycerides VLDL‐1

Cholesterol VLDL‐2

Free Cholesterol VLDL‐2

Phospholipids VLDL‐2

Triglycerides VLDL‐2

Cholesterol VLDL‐3

Free Cholesterol VLDL‐3

Phospholipids VLDL‐3

Triglycerides VLDL‐3

Cholesterol VLDL‐4

Free Cholesterol VLDL‐4

Phospholipids VLDL‐4

Triglycerides VLDL‐4

Cholesterol VLDL‐5

Free Cholesterol VLDL‐5

Phospholipids VLDL‐5

Triglycerides VLDL‐5

VLDL‐Apolipoprotein‐B100

VLDL Cholesterol

VLDL Free Cholesterol

VLDL Phospholipids

VLDL Particle Number

VLDL Triglycerides

mg/dL

mg/dL

mg/dL

mg/dL

mg/dL

mg/dL

mg/dL

mg/dL

mg/dL

mg/dL

mg/dL

mg/dL

mg/dL

mg/dL

mg/dL

mg/dL

mg/dL

mg/dL

mg/dL

mg/dL

mg/dL

mg/dL

mg/dL

nmol/L

mg/dL

1

1

1

1

1

1

1

1

1

1

1

1

1


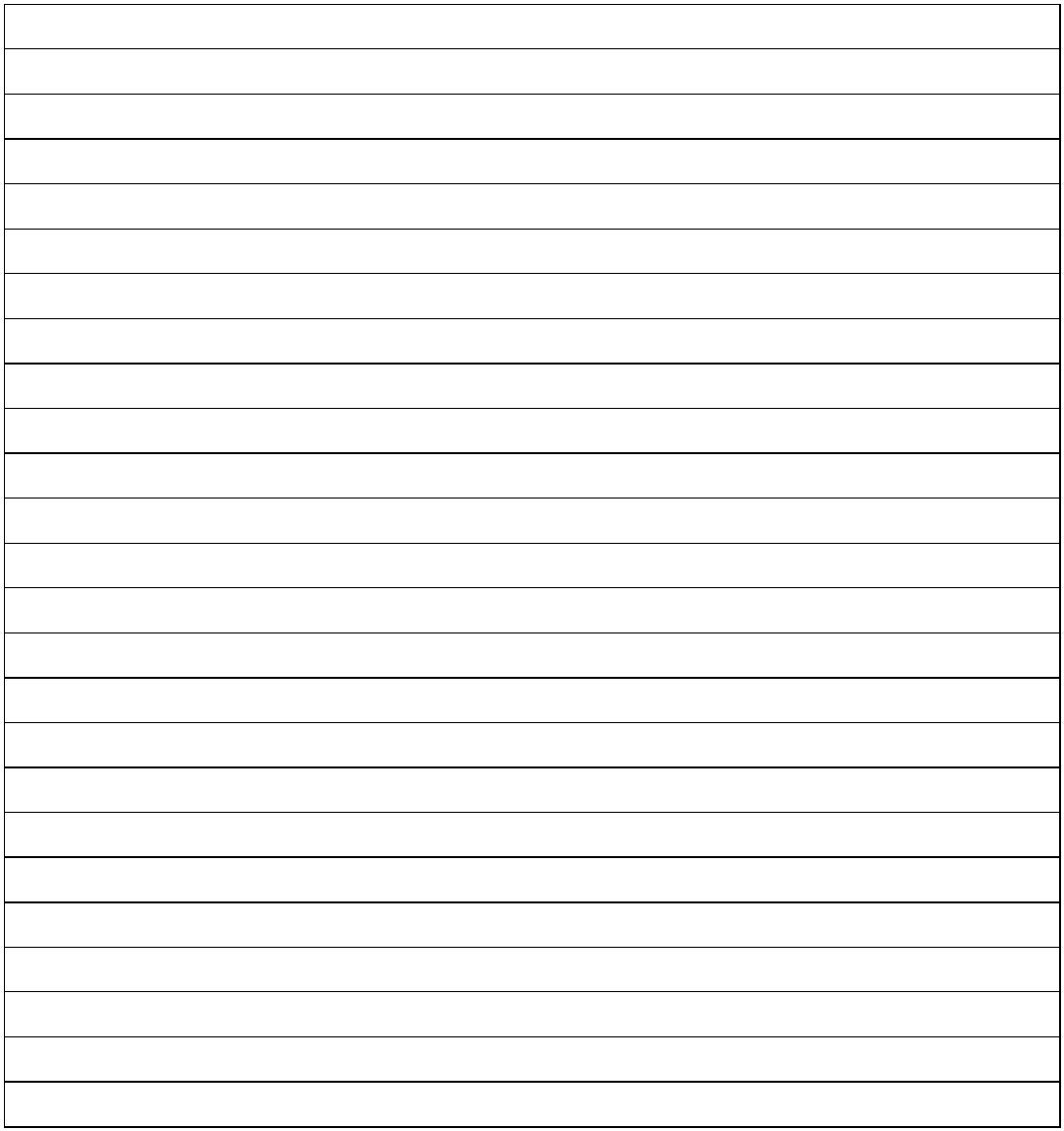

Supplement: Supplementary file 5 — Supplementary Material 5: Table S4B. Table S4B – FDR-significant cytokine-cytokine correlations in the endometriosis-only and sensitivity cohorts. [file 12916_2026_4999_MOESM5_ESM.docx]
